# Certificate of need laws: a systematic review and cost-effectiveness analysis

**DOI:** 10.1186/s12913-020-05563-1

**Published:** 2020-08-14

**Authors:** Christopher J. Conover, James Bailey

**Affiliations:** 1 Duke University Center for Health Policy and Inequalities Research, 310 Trent Drive, Durham, NC 27710 USA; 2 Department of Economics, Providence College, 1 Cunningham Sq, Providence, RI 02918 USA

**Keywords:** Certificate of need, Systematic review, Cost-effectiveness analysis, Hospital regulation

## Abstract

**Background:**

Certificate of Need (CON) laws, currently in place in 35 US states, require certain health care providers to obtain a certification of their economic necessity from a state board before opening or undertaking a major expansion. We conduct the first systematic review and cost-effectiveness analysis of these laws.

**Methods:**

We review 90 articles to summarize the evidence on how certificate of need laws affect regulatory costs, health expenditures, health outcomes, and access to care. We use the findings from the systematic review to conduct a cost-effectiveness analysis of CON.

**Results:**

The literature provides mixed results, on average finding that CON increases health expenditures and overall elderly mortality while reducing heart surgery mortality. Our cost-effectiveness analysis estimates that the costs of CON laws somewhat exceed their benefits, although our estimates are quite uncertain.

**Conclusions:**

The literature has not yet reached a definitive conclusion on how CON laws affect health expenditures, outcomes, or access to care. While more and higher quality research is needed to reach confident conclusions, our cost-effectiveness analysis based on the existing literature shows that the expected costs of CON exceed its benefits.

## Background

Certificate of need (CON) statutes were designed to hold down health costs by limiting unnecessary proliferation and duplication of health facilities, to improve quality by regionalization of selected types of surgical or other procedures where a volume-quality relationship exists and to improve access to care by preventing competitors from “cream-skimming” paying patients, leaving selected facilities with disproportionately high uncompensated care loads. The first CON statute was enacted in New York in 1964 and due to strong federal encouragement in the early 1970s, such programs were adopted by virtually all states by the early 1980s. Following the abandonment of federal support in the mid-1980s, 14 states fully repealed their CON laws, leaving 37 states and District of Columbia in which such programs were operational in 2008. Debate over CON has revived in recent years, with New Hampshire repealing its CON program in 2016 and Florida repealing most CON requirements in 2019.

### New contribution

Research has examined the impact of various dimensions of CON programs, but we are unaware of any published systematic review of this literature or any net assessment of the benefits and costs of these statutes. To evaluate the existing research, we performed a systematic review to address two key questions with several related parts. The questions are as follows:

**Key Question 1**: What are the regulatory costs of CON programs, including a) federal and state government regulatory costs and b) industry compliance costs?

**Key Question 2**: What are the key impacts of CON programs on a) health expenditures; b) health outcomes; and c) access to care?

We performed a database literature search that identified 1035 articles (939 unduplicated). Of these, we excluded 885 that clearly did not meet our inclusion criteria after abstract review. Of the 54 remaining articles subjected to full review, 13 were rejected and 41 retained. In addition to the database search, we solicited articles from systematic web searches and experts in the field. These sources provided 54 additional items, of which 49 were used in the preparation of the net assessment, yielding a full total of 90 articles.

We summarize the findings of this literature and use its findings to estimate the costs and benefits of CON laws. Cost-benefit analysis is generally used to convert disparate societal costs (such as increased spending or travel time) and benefits (such as reduced mortality) of a treatment or policy into dollar terms. The benefits are added together and the costs subtracted in order to provide a single estimate (or range of estimates) for how the policy affects overall societal welfare (wellbeing). See Viscusi [1] for a more detailed explanation and a defense of pricing health outcomes.

We estimate that the average cost-benefit ratio of CON is 1.08, meaning costs exceed benefits by 8%, with the average costs exceeding benefits by an estimated $302 million per year. However, these estimates are quite uncertain given that the literature on how CON affects key outcomes such as mortality and spending is quite mixed.

### History

The history of CON is well-documented elsewhere, but as a general proposition, state policy currently has been moving in the general direction of eliminating CON or softening its stringency since the 1980s [2]. Simpson [3] provides an exhaustive early history of CON; Conover and Sloan [2] provide an update of CON trends since the early 1980s.

CON regulation first began in New York in 1964. The *National Health Planning and Resource Development Act of 1974* (P.L. 93–641) provided federal funds for state efforts to implement CON and proposed severe financial penalties for failure to do so. Due to repeated congressional postponement of effective dates, the latter provisions never were put into effect, but the prospect of these rules being implemented induce virtually every state to make concerted efforts to comply [3]. P.L. 93–641 was repealed in 1986. Over the subsequent decade, 20 states have elected to drop their CON programs for acute care services (6 of these retained CON for nursing homes or other long term care services).

There are no longer any federal regulations governing CON. The American Health Planning Association [4] puts out an annual report that codifies by state the key features of all current CON programs (e.g., the dollar value of review thresholds, scope of services subject to review), as well as Web links to all operational programs where further details on pertinent regulations may be found.

### Key elements

CON programs generally establish dollar thresholds for review of proposed projects related to new building or expansion of health services. Some states set different dollar thresholds (generally lower) for long term care facilities than for other acute care facilities such as hospitals. In addition, the thresholds for equipment generally are lower for equipment such as lithotripters than for capital projects; in the most stringent states, all projects involving equipment of a particular type are reviewed regardless of the size of the project. Likewise, states sometimes draw a distinction between new services as opposed to expansion of existing services. All in all, there is wide variation in the scope and mechanics of CON review across states (see AHPA 2009 [5] for a detailed breakdown).

As of 2008, there were 31 states (including District of Columbia) with CON for both hospitals (and other acute care services) and nursing homes, while another 6 states have retained CON for nursing homes only [5].

Historically, CON was enforced by the threat of disallowing Medicare or Medicaid payment for facilities or services that had not undergone CON approval. Since repeal of these provisions, states have adopted a variety of mechanisms for enforcing CON regulations. States generally can enforce CON requirements by denying, suspending or revoking the license or certification of a facility not in compliance, but in addition, some states impose sizable administrative penalties (e.g., $5 million) for specific violations of CON statutes.

## Methods

We investigated two broad research areas related to the impact of CON regulation in the U.S. The questions are listed below, along with a brief description of our analytical approach.

### Regulatory costs of CON regulation

**Key Question 1a.** *What is the amount of government regulatory costs related to the CON regulation?* This includes state costs to monitor and enforce rules related to certificate of need for hospitals, nursing homes or other facilities to which CON is applicable.

**Key Question 1b.** *What is the amount of industry compliance costs related to CON regulation?* This includes all administrative costs and enforcement penalties borne by private, state or locally owned health facilities subject to state CON rules. Monetary penalties may be viewed as a transfer, but the remaining costs represent real resource losses to society.

### Major impacts of CON regulation

**Key Question 2a.** *What is the net impact of CON regulation on health expenditures?* Historically, CON policy was justified on market-perfecting grounds to overcome the weak incentives for economic discipline resulting from a combination of cost-based reimbursement and pervasive third-party payment for health care. According to this theory, CON could enhance efficiency by regionalizing expensive tertiary facilities and preventing the costly duplication of technology or facilities. Skeptics argue that CON is a form of industry protection from competition, pointing out that states that first adopted CON were more likely to have more hospital beds and lower occupancy rates. Reduced competition could have adverse effects on health expenditures (by allowing facilities to charge higher prices). Therefore, our search allowed for the possibility that CON could decrease, increase or have no impact on health expenditures.

**Key Question 2b.** *What is the impact of CON regulation on health outcomes?* To the extent that facilities with higher volumes of selected procedures have better outcomes, [6] regionalization resulting from CON could have a corollary benefit in the form of improved patient outcomes. Likewise, to the extent that CON efforts to prevent “cream-skimming” were successful, this might allow the survival of certain facilities such as large urban public hospitals that might otherwise be forced to shut down for lack of sufficient paying patients. In theory, this too could result in health benefits and/or reductions in avoidable hospitalizations if indigent patients were able to receive essential care on a timely basis. But limitations on competition also have the potential to result in lower quality care, so we sought literature that related CON to outcomes in either direction. Changes in either morbidity or mortality could be monetized using conventional methods.

**Key Question 2c.** *What is the impact of CON regulation on access to care?* Another rationale for CON is to ensure access to disadvantaged populations or to maintain provider financial margins to allow them to cross-subsidize indigent care, i.e., help offset uncompensated care costs [7]. Whether informally or formally through explicit commitments required for approval, CON regulators have the power to restrict approval to facilities willing to supply services perceived to be in the public interest, such as charity care or care in medically underserved areas [8]. Even if they had no measurable impact on health outcomes, such improvements in access to care would be of value, so we sought to ensure to include literature focused on this dimension of CON performance. However, some have raised concerns that CON may restrict access to care through output restriction and market division. That is, the CON process may allow regulated hospitals or facilities to “carve out” the distribution of patients, beds or suites (both geographically or by specialty niche). “Output restriction and market division are two classical tactics that are used by economic oligopolies to manage supply and create market power”. ([8] , p.1085) Such practices may be tacitly encouraged through CON programs. Therefore, our search allowed for the possibility that CON could decrease, increase or have no impact on access to health services.

#### Literature search and review

We searched the MEDLINE, CINAHL, Lexis-Nexis, and Public Affairs Information Service (PAIS), ISI Web of Knowledge, ProQuest Dissertations & Theses, and *Health Affairs* databases through 2010. A professional librarian conducted each search, customizing the searches for each research question. We present our full search strategy for MEDLINE in Appendix B as an example of our process. In general, our search strategy is similar to that of Conover and Wiechers [9].

In addition to our searches of formal databases, we searched the websites of health policy firms, research organizations, and foundations for relevant articles; the only relevant articles from this search came from the American Health Planning Association and the Employee Benefits Research Institute.

### Study selection

We excluded studies with no original data, no outcomes of interest, studies superseded by an updated version, studies published in abstract form only, and studies using only pre-1975 data.

### Further detail

A detailed evidence table documenting all the methods and sources used in the net assessment appears in Appendix A. Appendix B codifies all the search terms used for each database examined. We did not use a registered protocol.

## Results

We report the results in two main sections here and then turn to a net assessment assembled using all the available evidence. A summary table and graph are contained at the end of this section.

### Results of literature search

The literature search identified 1035 articles (939 unduplicated). Of these, we excluded 885 that clearly did not meet our inclusion criteria after abstract review. Of the 54 remaining articles subjected to full review, 13 were rejected and 41 retained. In addition to the database search, we solicited articles from systematic web searches and experts in the field. These sources provided 54 additional items, of which 49 were used in the preparation of the net assessment. One investigator extracted information from each article into evidence tables. Another investigator independently assessed the accuracy and completeness of the literature review and the net assessment developed using evidence from the synthesis.

### Literature quality

In contrast to the dearth of studies available on other aspects of health regulation, there is a comparative embarrassment of riches in the area of CON, as it was the first major state-level initiative to curtail health spending. Kessler and McClellan [10] show that in the 1980s, the welfare effects of hospital competition were ambiguous, but in the 1990s hospital competition unambiguously improved social welfare, i.e., lowering average expenditures per patient and mortality among Medicare patients being treated for heart attacks in the 1990s. Thus, in cases where evidence is conflicting, more recent evidence about CON’s effects has been given greater weight than earlier studies.

Another important consideration in evaluating the quality of a study’s results is whether it addresses the issue of endogeneity or omitted variables bias. Researchers generally try to control for known differences between the states that might have an influence on an outcome variable of interest. But they can never be certain they have taken into account every unobserved difference that might matter. We put greater weight on studies that use methods to address these problems, such as state fixed effects, difference-in-difference analysis, or two-stage least squares.

### Key question 1: regulatory costs of CON regulation

#### Public administration

KQ 1a concerned the effects of CON regulation on the costs of public administration. We found no nationwide estimate of such costs; however, total staffing for CON agencies by state are reported in a 1986 DHHS report [11].

#### Compliance costs

KQ 1b concerned the costs of compliance with the Act. While we found sporadic allusions to costs incurred by CON applicants and/or delays imposed by the CON process, we found no systematic literature summarizing such costs or providing reliable parameters from which to construct a national estimate.

### Key question 2: major impacts of CON regulation

#### Impact on health expenditures

KQ 2a concerned the Act’s effects on health spending. Because of the volume of available literature, we have divided it based on the category of facilities to which CON applies (hospitals, nursing homes and other) and discuss individual papers in detail only in Appendix C.

## Hospital CON

The evidence regarding hospital CON’s effect on health expenditures is generally mixed, although one could credibly conclude that the weight of this evidence is that CON has no impact on health costs overall. Because of the sheer number of studies available, including several of high quality that examine CON’s impact on overall health expenditures, we have excluded many other narrowly focused studies that examined CON’s effects on cost per day or cost per stay since one cannot reliably extrapolate from these results to a global cost effect (see Conover and Sloan [2] for a review). Some more recent studies [2, 12]. have found that CON produces a savings in the hospital sector while having no detectable impact on health expenditures overall. In addition, most early studies did not correct for the endogeneity of CON regulation. More recent studies have corrected this oversight by using either two-stage models to estimate demand for regulation (e.g., [13]) or using state-level fixed effects estimators, (e.g. [2, 12, 14]).

## Nursing home CON

We found at least a half dozen studies that examined the effects of nursing home CON on bed capacity, but most of these are at least two decades old and not directly informative of the net effect on health spending (since even demonstrated reductions in nursing home bed supply may simply spill over into expenditures on home and community-based substitutes). However, two more recent studies explicitly examined the impact of nursing home CON on health expenditures, with mixed results. We discuss these in detail in Appendix C.

## Other types of CON

The literature on how other types of CON affect spending is relatively thin. Three papers, discussed in Appendix C in detail, provide suggestive evidence that hospice CON, home health CON, and dialysis CON increase spending, but these papers generally do not use direct measures of spending or costs.

### Impact on health outcomes

KQ 2b concerned the effects of CON regulation on health outcomes. The Federal Trade Commission and U.S. Department of Justice, [15] after extensively reviewing the available literature and hearing from expert witnesses, concluded the following: “The Agencies believe that CON programs are generally not successful in containing health care costs and that they can pose anticompetitive risks. As noted above, CON programs risk entrenching oligopolists and eroding consumer welfare. The aim of controlling costs is laudable, but there appear to be other, more effective means of achieving this goal that do not pose anticompetitive risks *A similar analysis applies to the use of CON programs to enhance health care quality and access*” (emphasis added; ch. 8, p. 5). There are three bodies of CON literature that address health outcomes: the largest group examines CON’s impact on mortality, a much smaller body examines other health outcomes and the third focuses on nursing home quality.

## Mortality losses

The CON literature related to mortality losses has been the most disparate, with three studies finding that CON reduces mortality, five showing no effect, and three finding that CON increases mortality. We discuss these studies in detail in Appendix C. Oddly, almost the entire literature on CON and mortality has focused on heart surgery mortality; only Shortell and Hughes [16] consider other procedures, finding that mortality is higher in states with stringent CON regulation.

## Other health outcomes

A handful of studies have examined health outcomes unrelated to mortality risk, suggesting that CON worsens the quality of dialysis care [17] and has mixed effects on the quality of cardiac care [18, 19]. We discuss these studies in more detail in Appendix C.

## Nursing home quality

There is a companion literature on the impact of nursing home CON on quality measured in terms of structure, process and outcome indicators.

In theory, a binding CON policy provides no incentive to nursing homes to compete for Medicaid residents on the basis of quality, the theory argues that under such a binding constraint a higher payment level actually leads to lower quality. It also can lead to access problems for Medicaid patients such as “cream-skimming” patients likely to require less intensive nursing home services. A large body of literature--excellently reviewed in Caldwell [19]--examines evidence related to this theory (and to “cream-skimming” and other access barriers faced by prospective nursing home patients on Medicaid), but recent evidence in a series of studies by David Grabowski [20–24] suggests higher Medicaid payments do result in higher nursing home quality, contradicting a raft of earlier results by Nyman [25–28]. Nursing home occupancy rates have declined over time, suggesting less “excess demand.” This in turn suggests that either nursing home CON may impose less of a binding constraint, or that substitutes for nursing home care have arisen that dissipate some of the adverse consequences that nursing home CON might historically have imposed.

More directly though, Zinn [29] showed that the presence of a statewide nursing home construction moratorium is associated with a lower level of quality of care, measured in terms of both lower RN staffing and a higher percentage of residents who are physically restrained.

### Impact on access to care

KQ 2c concerned the effects of CON regulation on access to health care. As of 1994, most CON programs required facilities to provide a “reasonable amount” of care to the poor [30]. The literature on the actual effects of CON regulation on access have been mixed. Nine studies found a positive effect of CON on access, two found no effect, and sixteen found a negative effect. We discuss these studies in more detail in Appendix C. The greatest challenge in summarizing the literature on access to care is that almost every study defines “access” in a different way (amount of care overall, amount of uncompensated care, travel time to care, racial disparities in care, et cetera).

## Cost-effectiveness analysis

### Overview

During this systematic review, we identified a large body of literature addressing the regulatory costs and various impacts of CON regulation on health expenditures, health outcomes and access to care. Our review systematically identified, organized, and critically analyzed the relevant studies. While we located several in-depth qualitative reviews of various components of this literature, [12, 31, 32] none has been as thorough and systematic as this one. More importantly, no prior studies have reported an aggregate “bottom line” assessment of the nationwide benefits and costs of CON regulation. Consequently, in what follows, we have synthesized the best available evidence in order to provide such a net assessment. The wide range of evidence contained in the research surrounding certificate of need policies provides mixed results, showing that CON may either save or cost both money and lives.

### How benefits and costs were calculated

We have calculated the regulatory costs in the following fashion (lower- and upper-bound estimates are shown in parentheses: full details of methods and sources are in Table A1).

#### Government regulatory costs

We multiply average CON staffing per state in 1986 [11] times the number of states with CON in 2008 and multiply this by the average total compensation of state employees in 2008. ([33], p. Table 843) Since we do not know whether CON employees have total compensation that is lower or higher than the average for all state employees, we use +/− 25% for upper and lower bounds.

#### Industry compliance cost

Lacking a firm national estimate, we calculated CON compliance spending per $10,000 personal health care expenditures (PHCE) in 2013 using data from the State of Washington [33, 34] and adjusting this figure to 2008 dollars using the change in U.S. PHCE between 2008 and 2013 [35]. The Lewin Group ([36], p.12) characterizes the Washington program as representing a “middle of the road” approach to CON, so no further adjustment to this figure appeared necessary. We applied this estimate to the total amount of personal health care spending in states with CON [5, 37, 38].

*Key Impacts: Health Expenditures.* We estimated three separate health spending effects.

*Reduction in Coronary Artery Bypass Grafting (CABG) Facilities*. We calculated the number of new CABG programs averted per 1000 CABG patients using Kolstad’s [39] estimates from Pennsylvania. We applied this to the estimated number of CABG surgeries in states with CON (allocating total CABG surgeries in 2008 [35] based on the distribution of total adults age 18 and older [40]. We multiplied the result times the amortized annual capital cost per CABG cost; this was derived from total capital costs reported in the literature, [41, 42] adjusted for inflation using the Turner Building Cost Index, [43] using the same 15-year amortization period as Cutler et al. (2010), and the prevailing bank prime loan rate (5%) as of July 2008 [44].

*Medicare Spending (Stringent CON States)*. We estimated the reduction in Medicare spending in stringent CON states using Sloan and Conover’s [45] estimate of 1.8%, applying this to estimated Medicare spending in states with stringent CON in 2008 [37].

*All Other Health Spending*. In light of all the varied findings regarding CON on total expenditures, no reduction in overall spending seems most consistent with the available evidence. The 13.6% reduction in spending reported in Lanning, Morrisey and Ohsfeldt [13] is both very dated and inconsistent with subsequent studies by Sloan and Conover [45]--using an updated version of the same data series and similar methods for controlling for endogeneity--showing no statistically significant effect of CON (or even stringent CON) on overall health spending. But the same study shows there are savings to Medicare in stringent CON states. Moreover, the Sloan and Conover analysis was performed using data through 1998; hence, it hypothetically should have picked up any beneficial effects of CON on CABG spending. The only way to reconcile these results is to assume that Medicare and CABG savings are offset by corresponding increases in health spending elsewhere, resulting in zero overall net measured impact. Based on estimating aggregate personal health spending in CON states using CMS estimates of per capita spending by location of provider [37]. This implies an expected increase in all other health spending in CON states of 0.19%.

*Home Health Cost Increases*. We elected not to use the Anderson and Kass [46] finding of a slight increase in Medicare-certified home health agency costs due to CON both because the finding is so dated and has so many methodological limitations.

*Key Impacts: Access to Care, Patient Time Losses*. We used Kolstad’s estimate of $7.50 per CABG patient in time losses related to restricted supply of CABG facilities under CON (no further adjustment was made since it is based on Pennsylvania’s hourly wage, which is nearly identical to the national average [47]. This was multiplied by total CABGs in states with CON in 2008.

*Key Impacts: Health Losses*. We calculated four separate effects on mortality.

*General Elderly Hospital Mortality Rates, Stringent CON States*. Because it measured effects using data now nearly 2 decades old and also has its own methodological limitations, the Shortell and Hughes [16] finding that mortality was 6% higher than expected in states with stringent CON was assigned a weight of 25% (consistent with how other mortality estimates of that vintage were treated) and used as the 99th percentile cost estimate. This increase is applied to all elderly hospitalization deaths for the elderly in states with stringent CON (estimated by applying a national hospital death rate for the elderly [48] to total reported elderly hospitalizations by state [49]. This estimated mortality increase is monetized using an adjusted value of statistical life for the elderly in 2008 [50] that takes into account their lower life expectancy relative to the general population (i.e., this calculation assumes the elderly place the same value on an added year of life as the general population, but this means the aggregate value they place on mortality risk reductions on a per life saved basis is lower since they have fewer years of life remaining).

*CABG Mortality, All Patients*. The expected number of deaths averted due to CON is calculated using the estimated total CABGs in states with CON, 2008 times the weighted average reduction of 1.1 deaths per 1000 patients due to CON (detailed in Table A2 using results from Ho, [51] DiSesa et al., [52] and Robinson et al. [42] This estimated mortality reduction is monetized by first multiplying the estimated added life expectancy per CABG patient (8 years [53]) times the average quality of life of a typical CABG survivor (0.9 on a scale of death [0] to perfect health [1 [54];]) to obtain the net increase in quality-adjusted life years (QALYs) attributable to CON. The monetary value of these mortality gains is calculated by multiplying the number of QALYs times a willingness-to-pay estimate of the value of a QALY ($239,000 [50]).

*CABG Mortality, Medicare Patients (Stringent CON States)*. The expected number of deaths averted due to stringent CON is calculated using the estimated total CABGs in states with stringent CON, 2008 times the weighted average reduction of 7.3 deaths per 1000 elderly patients due to stringent CON (detailed in Table A2 using Popescu et al. [55]). Each death averted is monetized as described immediately above except that the added life expectancy per CABG patient is adjusted to reflect the shorter life expectancy for elderly CABG patients [56].

*CABG Mortality, Medicare Patients (Non-Stringent CON States).* The expected number of deaths attributable to non-stringent CON is calculated in the identical fashion by authors using the weighted average increase of 4.9 deaths per 1000 elderly patients (detailed in Tables A1 and A2 using Vaughn-Sarrazin et al. [57] Popescu et al. [58], DiSesa et al. [52] Popescu et al. [55] and Ho et al. [59].

*Social Welfare Losses: Efficiency Losses from Tax Collections.* We accounted for the marginal overhead costs—which account for government collections costs, taxpayer compliance costs, and marginal excess burden (deadweight losses)—of state taxes by multiplying state administration costs times 40.5% (Duke University Center for Health Policy and Inequalities Research (CHPIR, 2015a). By the same logic, every dollar of Medicare savings is associated with companion “hidden” savings to society from the marginal overhead costs of federal taxes. Hence, all Medicare savings have been multiplied by 48.2% to account for such gains.

*Social Welfare Losses: Efficiency Losses from Regulatory Costs.* All industry compliance costs are presumed to be roughly equivalent to a sales tax, i.e., raising prices and reducing demand/output correspondingly. We therefore multiply the sum of industry compliance costs and health expenditure changes times the marginal excess burden associated with sales taxes (23.3%) [60].

### Summary findings

The results are summarized in Table 1. The expected benefits of CON regulation in 2008 ($3.6 billion) are exceeded by its costs ($3.9 billion) by $302 million. Put another way, its benefits are 8% less than its costs (conversely, its expected costs exceed expected benefits by 8%). There is considerable uncertainty surrounding all these figures.

**Table 1:** Benefits and Costs of Certificate of Need (millions of 2008 dollars)

		DISTRIBUTION			
	EXPECTED	Lower bound (5%)	25%	75%	Upper bound (95%)
Benefits	$ 3647	$ 1658	$ 2791	$ 4471	$ 5821
Costs	3949	932	1965	5185	9380
Net Costs	302	(3556)	(1754)	1768	5987
Benefit: Cost Ratio	0.92	0.30	0.65	1.86	4.08
Cost-Benefit Ratio	1.08	0.24	0.54	1.57	3.34

Probability benefits exceed costs: 54%

**Fig. 1:**
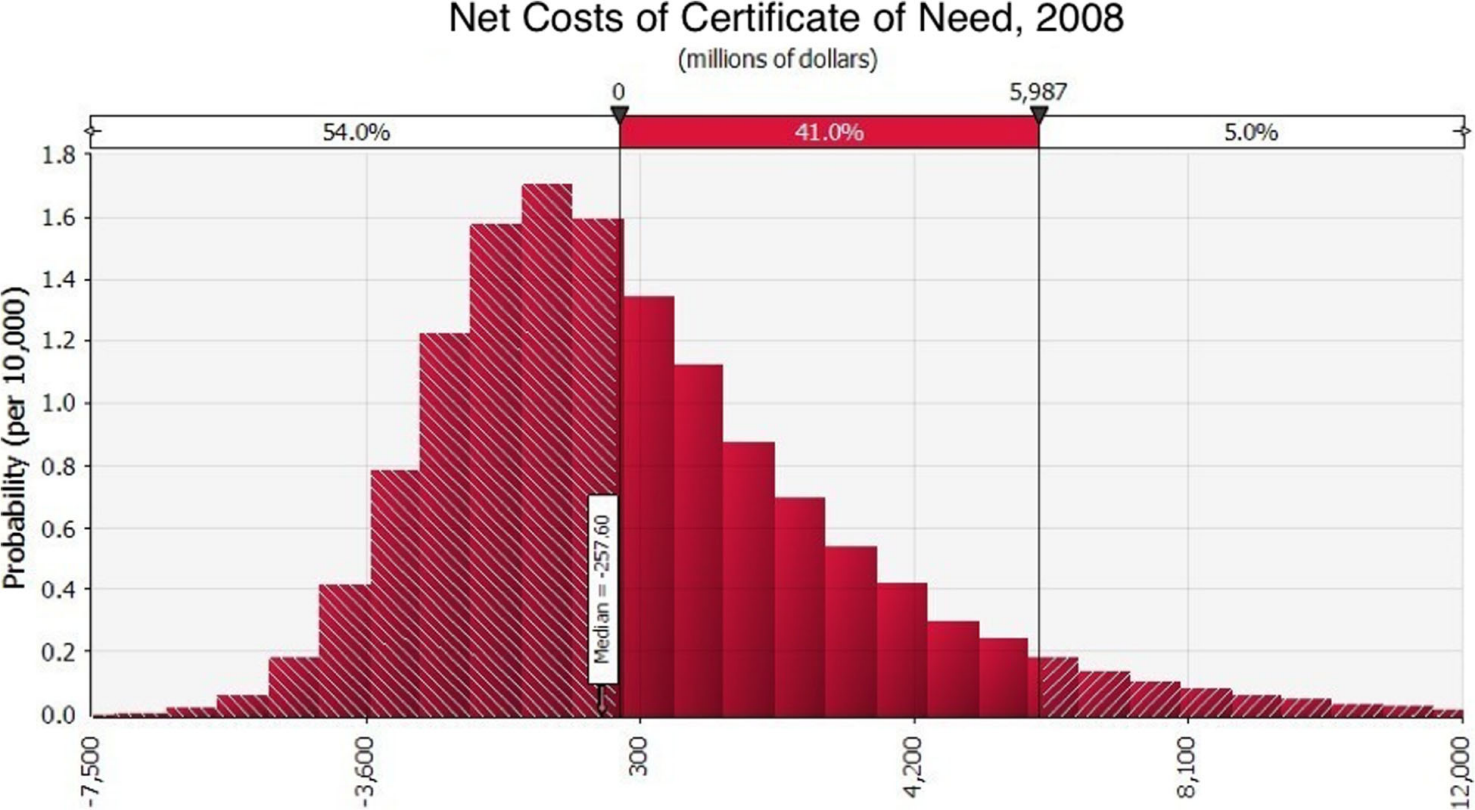
Net Costs of Certificate of need, 2008

Consequently, as shown in Fig. 1, the weight of the evidence suggests that CON creates more costs than benefits. We estimate that the probability that benefits exceed costs is 54%. However, because net costs are skewed in the direction of reducing rather than increasing social welfare our best estimate is that social welfare would increase by several hundred million dollars a year if CON were repealed in the 37 states that retain it, or if it were modified in some fashion to either increase benefits, reduce the costs of achieving them or some combination. It is beyond the scope of this synthesis to make recommendations about how the latter might be achieved.

### Limitations

The information compiled in this report may permit policymakers to identify areas in which regulatory costs appears excessive relative to benefits. However, this report is *not* designed to provide specific guidance on ways in which the objectives of CON restrictions might be pursued more cost-effectively.

Our cost-effectiveness analysis is limited by the quality and scope of the literature upon which it depends. In particular, the literature on CON and mortality focused almost entirely on heart surgery mortality, and so this provides most of the evidence weight for our mortality estimates even though it represents only a small fraction of all mortality. Our estimates of the effect of CON would likely be larger if more broad mortality measures were available (either larger costs or larger benefits depending on whether this literature found CON to increase or reduce mortality). Going forward, we hope researchers will study the effect of CON on types of mortality other than just post-heart-surgery. The literature on CON and access to care, by contrast, is too varied, with almost every study measuring “access to care” in a different way. We used only patient travel time in our cost-effectiveness analysis, as the measure that was easiest to translate into dollar terms, but it is certainly not the only measure of access and arguably not the best. The effect of CON on travel time was a small cost, but other access measures may have yielded either larger costs or benefits. Finally, the literature on the regulatory and compliance costs of CON was sparse and out-of-date.

In addition to the limitations of this overall body of literature and the particular challenges it poses, our review process also had some limitations. Because of time and resource constraints, we did not conduct dual, independent, blinded review of articles for inclusion or abstraction of information into evidence tables. We also did not rely on a formal scoring process for grading the quality of individual articles. Our review also stopped in 2010, leaving room for future work to focus on the most recent literature. Newer literature may find systematically different results, not only due to new econometric techniques or areas of focus, but because the true underlying effect of CON may change over time (in particular given insurance-market reforms; see Bailey [61]).

However, we have labored to be as transparent as possible in demonstrating to readers the methods, sources and analytic assumptions used to develop the net assessment. This approach provides an exceptionally high degree of granularity, allowing skeptical readers to substitute their own assumptions to calculate an alternate result. As well, we have made a point of codifying the uncertainty inherent in all of our estimates rather than relying simply on point estimates. While there may be components of benefits or costs that we have missed or inaccurately measured, we have no reason to believe that the process we used would have systematically biased our estimate of net costs in any obvious direction.

## Conclusion

Based on the available evidence, CON programs appear to have achieved some benefits. However, the costs imposed such programs, including regulatory costs as well as adverse effects on health spending, exceed those benefits by an estimated $302 million a year. On average, the cost-benefit ratio is 1.08, meaning costs exceed benefits by 8%. Consequently, the weight of the evidence suggests that CON creates more costs than benefits. However, our estimates are quite uncertain; we have 90% confidence the true value of the cost-benefit ratio lies between 0.30 and 4.08.

We estimate that the probability that CON’s benefits exceed its costs is 54%. However, because net costs are skewed in the direction of reducing rather than increasing social welfare, our best estimate is that CON decreases social welfare by several hundred million dollars per year. These effects are largely driven by health expenditures and health status; the literature yields central estimates that CON has no effect on expenditures and slightly improves health, but suggests that CON is more likely to lead to large negative effects (more spending and worse health) than large positive ones (less spending and better health). Therefore, expected social welfare would increase if the 35 states that continue to maintain CON programs repealed them or modified them in some fashion to either increase benefits, reduce the costs of achieving them, or some combination.

### Supplementary information

**Additional file 1.**

## Data Availability

As a review article, we do not have data or material to share beyond what is shown here in our tables, references, and appendices.

## Appendix A

### Evidence tables

**Table 2:** Costs and Benefits of Certificate of Need Regulation (millions of 2008 dollars)

COST CATEGORY	COSTS			BENEFITS			NOTES
	Expected	Lower bound	Upper bound	Expected	Lower bound	Upper bound	
**Government Regulatory Costs**	**20**	**9**	**31**	**–**	**–**	**–**	
Federal	–	–	–	–	–	–	
State	20	9	31	–	–	–	[A]
**Compliance Costs**	**146**	**109**	**182**	**–**	**–**	**–**	
Administrative costs	146	109	182	–	–	–	[B]
**Key Impacts**	**3272**	**677**	**7836**	**2786**	**1291**	**4418**	
Health expenditures	2023	104	6044	2023	848	3229	
Reduction in CABG facilities	–	–	–	456	233	711	[C]
Medicare spending	–	–	–	1566	416	2748	[D]
All other health spending	2023	104	6044	–			[E]
Patient time losses	2	1	3				[F]
Health status	1247	314	3746	764	(1)	1974	
Morbidity losses	–	–	–	–	–	–	
Mortality losses	1247	132	3541	764	(1)	1974	
General elderly hospital mortality	1027	44	3303	–			[G]
CABG mortality, all patients				463	(199)	1455	[H]
CABG mortality, Medicare patients, stringent CON states				300	(4)	807	[I]
CABG mortality rates, Medicare patients non-stringent CON states	220	(97)	701				[J]
**Social Welfare Losses**	**512**	**66**	**1453**	**861**	**295**	**1511**	
Efficiency losses from tax collection	8	4	13	755	192	1400	[K]
Efficiency losses from regulatory costs	504	58	1446	106	54	168	[L]
**GRAND TOTAL**	**3949**	**932**	**9380**	**3647**	**1658**	**5821**	
**NET BURDEN (COSTS MINUS BENEFITS)**	**302**	**(3556)**	**5987**	**Probability that benefits exceed costs:**			**54%**

Update: 5/16/2015

**Note:** All lower and upper bounds are calculated using Latin Hypercube simulations in @RISK. Therefore, sub-totals generally are smaller than the sum of their components. All reported simulation results are based on 100,000 iterations. For simplicity, both costs and benefits are shown as positive numbers. However, costs are equivalent to negative benefits and vice-versa. Cases in which negative numbers appear under either costs or benefits illustrate instances in which an impact is not statistically significant using conventional standards: that is we are not 95% certain that the estimated impact does not fall above or below zero.

**Notes:**

[A] The expected value figure is calculated by authors: (Average FTE CON Employees in States with CON [P2]) x (Number of States with CON in 2008 [P1]) x (Average Total Compensation of State Employees in 2008 [P3]). Since it is uncertain whether CON employees have total compensation that is lower or higher than the average for all state employees, +/− 25% are used for upper and lower bounds.

[B] The expected value figure is calculated by authors: (CON Compliance Spending per $10,000 PHCE [P8]) x (PHCE Spending in CON States, 2008 [P11]) /10,000. Compliance spending per $10,000 personal health care expenditures (PHCE) are based on the average cost per hospital CON application in Washington state in 2013 [P4] adjusted for inflation to derive a 2008 figure, multiplied times the average annual number of CON applications in Washington state [P5] divided by PHCE in Washington state in 2008 [P11]. The result is divided by 100 to obtain CON cost per $10,000 PHCE.

[C] The expected value figure is calculated by authors: (Total CABGs in States with CON, 2008 [P21]) x [(New CABG Programs Averted per 1000 CABG Patients [P17])/1000) x (Amortized Annual Capital Cost per CABG Program, 2008 [P19]).

[D] The expected value figure is calculated by authors: (Share of 2008 Medicare Spending in States with Stringent CON [P13]) x [(Medicare Expenditures in 2008 [P12]) - (Medicare Expenditures in 2008)/ (1 - (Reduction in Medicare per Eligible Spending in Stringent CON States [P14])], using the 1.8% reduction in Medicare spending per eligible due to CON reported in Sloan and Conover [45].

[E] In light of all the varied findings regarding CON on total expenditures, no reduction in overall spending seems most consistent with the available evidence. The 13.6% reduction in spending reported in Lanning, Morrisey and Ohsfeldt [13] is both very dated and inconsistent with subsequent studies by Sloan and Conover [45] using an updated version of the same data series and similar methods for controlling for endogeneity showing no statistically significant effect of CON (or even stringent CON) on overall health spending. But the same study shows there are savings to Medicare in stringent CON states. Moreover, the Sloan and Conover analysis was performed using data through 1998, hence should have picked up any beneficial effects of CON on CABG spending. The only way to reconcile these results is to assume that Medicare and CABG savings are offset by corresponding increases in health spending elsewhere, resulting in zero overall net measured impact. This implies an expected increase in all other health spending in CON states of 0.19% [P16]. The expected value figure is calculated by authors: [(All Other PHCE Spending in CON states, 2008 [P15]) -(All Other PHCE Spending in CON states, 2008)/ (1 + (Increase in All Other PHCE Spending Due to CON [P16])].

[F] The expected value figure is calculated by authors: (Time Losses per CABG Patient Due to Restricted Supply [P20]) x (Total CABGs in States with CON, 2008 [P21]) /1000,000. The expected value is $7.50 per patient, as reported in [53] based on the median wage in Pennsylvania.

[G] Because it measured effects using data now nearly 2 decades old and also has its own methodological limitations, the Shortell and Hughes [16] finding that mortality was 6% higher than expected in states with stringent CON [P24] was assigned a weight of 25% (consistent with how other estimates of that vintage were treated) and used as the 99th percentile cost estimate. This increase is applied to all elderly hospitalization mortality for the elderly in states with stringent CON. Excess deaths due to stringent CON = (Elderly Hospital Patients, 2008 [P22]) x (Elderly In-Hospital Mortality Risk, 2008 [P23]) x (Increase in Medicare Mortality Rates Due to Stringent CON [P24]). This estimated mortality increase is monetized using an adjusted value of statistical life for the elderly in 2008 [P25] that takes into account their lower life expectancy relative to the general population (i.e., this calculation assumes the elderly place the same value on an added year of life as the general population, but this means the aggregate value they place on mortality risk reductions on a per life saved basis is lower since they have fewer years of life remaining).

[H] The expected number of deaths averted due to CON is calculated by authors: (Total CABGs in States with CON, 2008 [P21]) x (Change in CABG Mortality Rate/1000 Patients Due to CON [P26])/1000. This estimated mortality reduction is monetized by first multiplying the estimated added life expectancy per CABG patient [P27] times the quality of life of a typical CABG survivor [P28] to obtain the net increase in quality-adjusted life years (QALYs) attributable to CON. The monetary value of these mortality gains is calculated by multiplying the number of QALYs times a willingness-to-pay estimate of the value of a QALY [P29].

[I] The expected value is calculated by authors using the same logic described in footnote H (with one additional adjustment to account for elderly CABG life expectancy being shorter than for the average CABG patient): (Elderly CABGs in States with CON, 2008 [P30]) x (Change in Elderly CABG Mortality Rate/1000 Patients Due to CON [P31]) x (Added Life Expectancy per CABG Patient [P27]) x (Ratio of Elderly CABG Life Expectancy to Average CABG LE [P32]) x (Quality of Life, CABG Patients after 10 Years [P28]) x (Value of a QALY (thousands), 2008 [P29])/1,000,000.

[J] The expected value is calculated by authors using the same logic described in footnote I: (Elderly CABGs in States with CON, 2008 [P33] - Elderly CABGs in States with Stringent CON, 2008 [P30]D81) x (Added Life Expectancy per CABG Patient [P27]) x (Quality of Life, CABG Patients after 10 Years [P28]) x (Ratio of Elderly CABG Life Expectancy to Average CABG LE [P32]) x (Value of a QALY (thousands), 2008 [P29])/1,000,000.

[K] All cost figures are calculated by authors: (State Administration Costs) x (Marginal Overhead Cost, State Taxes [P37]). This accounts for the marginal cost of state tax collections inclusive of collections costs, compliance costs and deadweight losses (lost output) associated with state taxes. By the same logic, every dollar of Medicare savings is associated with companion “hidden” savings to society from the marginal overhead costs of federal taxes. Hence, all benefit figures are calculated as: (Medicare Savings, Stringent CON States) x (Marginal Overhead Cost, Federal Taxes [P36]).

[L] All losses borne by health industry are presumed to be roughly equivalent to sales taxes on revenues, i.e., raising prices and reducing demand/output. The marginal excess burden (MEB) is intended to measure the deadweight loss associated with such reduced output. All figures are calculated by authors: (Compliance Expenditures) x (Marginal Excess Burden, Sales Taxes [P38]).

**Table:**

Parameters:		DISTRIBUTION	@RISK FORMULA	EXPECTED	LOWER BOUND (5%)	UPPER BOUND (5%)	NOTES	
**Government Regulatory Costs**								
[P1]	Total states with CON, 2008	Constant	37	37	37	37	[a]	
[P2]	Average FTE CON employees in states with CON	Truncated Normal	10.2	10.2	5.1	15.3	[b]	
[P3]	Annual total compensation, state employees, 2008	Normal	52,058	52,058	39,044	65,073	[c]	
**Compliance Costs**								
[P4]	Average spending per CON application, WA, 2013	Constant	124,936	124,936	124,936	124,936	[d]	
[P5]	Average annual number of CON applications, WA	Constant	52	52	52	52	[e]	
[P6]	Personal health care expenditures (PHCE), WA, 2008 (millions)	Constant	$ 42,411	$ 42,411	$ 42,411	$ 42,411	[f]	
[P7]	Percentage change in PHCE, U.S., 2008–2013	Constant	17.9%	17.9%	17.9%	17.9%	[g]	
[P8]	CON compliance spending per $10,000 PHCE	Normal	$ 1.29	$ 1.29	$ 0.97	$ 1.61	[h]	
[P9]	Personal health care expenditures (PHCE), 2008 (000’s)	Normal	$ 1,997,199	$ 1,997,199	$ 1,964,445	$ 2,029,953	[i]	
[P10]	Share of PHCE in states with CON, 2008	Constant	56.6%	56.6%	56.6%	56.6%	[j]	
[P11]	PHCE spending in CON states, 2008 (millions)	Calculated	$ 1,130,663	$ 1,130,663	$ 1,112,100	$ 1,149,200	[k]	
**Key Impacts**								
Health expenditures								
[P12]	Medicare expenditures, 2008 (millions)	Normal	$ 440,758	440,758	$ 433,530	$ 447,987	[l]	
[P13]	Share of Medicare spending in states with stringent CON, 2008	Constant	19.2%	19.2%	19.2%	19.2%	[m]	
[P14]	Reduction in Medicare/eligible spending due to CON	Normal	1.8%	1.8%	0.5%	3.2%	[n]	
[P15]	All other PHCE spending in CON states, 2008 (millions)	Calculated	$ 1,045,988	$ 1,045,988	$ 1027,402	$ 1,064,578	[o]	
[P16]	Increase in per capita health spending due to CON	Exponential	0.19%	0.14%	0.01%	0.58%	[p]	
[P17]	New CABG programs averted per 1000 CABG patients	Normal	0.97	0.97	0.75	1.19	[q]	
[P18]	Capital cost per CABG program (millions)	Normal	$ 20.5	$ 20.5	$ 17.8	$ 23.2	[r]	
[P19]	Amortized annual capital cost per CABG program (millions)	Normal	$ 2.0	$ 2.0	$ 1.7	$ 2.2	[s]	
Patient time losses								
[P20]	Time losses per CABG patient due to restricted supply	Normal	$ 7.50	$ 7.50	$ 3.75	$ 11.25	[t]	
[P21]	Total CABGs in states with CON, 2008	Normal	238,586	238,586	131,404	267,020	[u]	
General elderly hospital mortality								
[P22]	Elderly hospital patients, 2008 (thousands)	Normal	13,902	13,902	11,504	16,300	[v]	
[P23]	Elderly in-hospital mortality risk, 2008	Normal	4.1%	4.1%	4.0%	4.2%	[w]	
[P24]	Increase in Medicare mortality rates due to stringent CON	Exponential	0.3%	7.9%	6.1%	9.7%	[x]	
[P25]	VOSL, elderly, 2008 (millions of dollars)	Triangle	$ 2.9	$ 2.9	$ 0.6	$ 5.9	[y]	
CABG mortality, all patients								
[P26]	Change in CABG mortality rate/1000 patients due to CON	Calculated	−1.1	−1.1	−2.8	0.6	[z]	
[P27]	Added life expectancy per CABG patient	Normal	8.0	8.0	6.0	10.0	[aa]	
[P28]	Quality of life, CABG patients after 10 years	Normal	0.9	0.9	0.7	1.1	[ab]	
[P29]	Value of a quality-adjusted life year (QALY), 2008 (thousands)	Triangle	$ 239.1	$ 239.1	$ 120.7	$ 407.0	[ac]	
CABG mortality, Medicare patients, stringent CON states								
[P30]	Elderly CABGs in states with stringent CON, 2008	Normal	43,840	43,840	24,145	49,065	[ad]	
[P31]	Change in elderly CABG mortality rate/1000 due to stringent CON	Calculated	−7.3	−7.3	−14.76	0.13	[ae]	
[P32]	Ratio of elderly CABG life expectancy to average CABG LE	Constant	0.54	0.54	0.54	0.54	[af]	
CABG mortality rates, Medicare patients non-stringent CON states								
[P33]	Elderly CABGs in states with CON, 2008	Normal	91,540	91,540	50,417	102,450	[ag]	
[P34]	Change in elderly CABG mortality rate/1000 in all CON states	Calculated	−0.9	−0.9	−2.0	0.1	[ah]	
[P35]	Change in elderly CABG mortality rate/1000, non-stringent CON	Calculated	4.9	4.9	−5.8	32.9	[ai]	
**Social Welfare Losses**								
[P36]	Marginal tax overhead costs, federal taxes	Normal	48.2%	48.2%	38.1%	64.3%	[aj]	
[P37]	Marginal tax overhead costs, state taxes	Normal	40.5%	48.2%	32.4%	53.9%	[ak]	
[P38]	Marginal excess burden, sales taxes	Normal	23.3%	48.2%	20.4%	26.1%	[al]	

**Parameter Notes:**

[a] States with CON in 2008 reported in [5]. Includes states with long-term care CON only as well as states in which CON regulations govern both acute care and long term care services.

[b] The expected value is a simple average of the number of employees working in CON programs per state in 1986 divided by the number of states participating in the program (40) in 1986 [11]. Due to uncertainty about how much that number has changed over more than two decades, the lower bound equals half the expected value. The upper bound figure is calculated as an output from @RISK simulation using a normal distribution (truncated at 1 on the presumption that any CON program would have at least 1 FTE employee) with the mean and lower bound shown.

[c] Figures equal annual total compensation for state government workers as reported in Table 643 of [62]. Since there is uncertainty about how much CON workers are paid relative to the average for all state employees, the lower-bound figure equals 25% below expected; upper-bound figure = 25% above expected.

[d] Washington state surveyed Washington hospitals in 2013 to estimate the administrative costs (staff time, legal and consulting fees) of a certificate of need application. The average reported cost was $84,236 in administrative costs, plus a $40,700 application fee [34].

[e] Washington state reportedly made decisions on 1786 CON applications between 1971 and July 2005 ([33] , p.3). The figure shown is the annual average.

[f] Figure calculated by author using CMS figures on state-level aggregate health spending estimates by provider of service for 2008 reported in [37].

[g] Figure calculated by author using CMS per capita health spending estimates for 2008 and 2013 reported in [35].

[h] The expected value is based entirely on the Washington state CON program, as calculated by author: [[(Average Spending per CON Application, 2013 [P4])/(1 + Percentage Change in PHCE, 2008–2013 [P7])] x (Average Annual Number of CON Applications)]/(PHCE, WA, 2008 [P6]). The Lewin Group characterizes the Washington program as having many similarities with the CON program in Illinois (which in turn is described as representing a “middle of the road” approach ([36] , p.12). This is consistent with the conclusion that “Washington is the 18th most regulated in the country” ([43], Fig. 2) (out of 37 programs that maintain CON regulation). Thus, it is reasonable to use data from the WA program as an expected value. However, given that the total number of CON applications includes those for nursing homes or other non-hospital facilities, while the cost per application is based solely on hospital costs, lower and upper bounds have been calculated as 25% deviations from the expected value.

[i] Figure shown is 2008 PHCE amount based on health spending by state of provider from the National Health Expenditure Accounts, as reported in [37]. NHEA data generally are based on establishment surveys and no measurement errors are formally reported. However, NHEA staff concede “clearly there is statistical uncertainty surrounding” the NHEA estimates [38], so lower and upper bounds were calculated assuming a relative standard error of 1%.

[j] Expected figure is calculated by authors using estimates health spending by state of provider from the National Health Expenditure Accounts, as reported in [37] and number of states with CON regulation in 2008, as reported in [5].

[k] Figures are calculated by authors: (PHCE, 2008 [P9]) x (Share of PHCE in States with CON, 2008 [P10]). Lower and upper bounds are calculated as outputs from @RISK simulation.

[l] Actual 2008 Medicare expenditures by state are reported in [35]. For reasons explained in footnote [jZ], lower and upper bounds were calculated assuming a standard error of 1%.

[m] Figures are calculated by authors from PHCE data by state reported in [37] using states reported in [63] as having stringent CON in 2008.

[n] The expected value is the estimated reduction in Medicare spending in states with stringent CON, as reported in [45], with lower and upper bounds calculated from the reported standard error.

[o] The expected value figure is calculated by authors: (PHCE Spending in CON States, 2008 [P11]) - (Medicare Expenditures, 2008 [P12]) x (Share of Medicare Spending in States with Stringent CON, 2008 [P13]).

[p] In light of all the varied findings regarding CON on total expenditures, no reduction in overall spending seems most consistent with the available evidence. The 13.6% reduction in spending reported in Lanning, Morrisey and Ohsfeldt [13] is both very dated and inconsistent with subsequent studies by Sloan and Conover [45] using an updated version of the same data series and similar methods for controlling for endogeneity showing no statistically significant effect of CON (or even stringent CON) on overall health spending. But the same study shows there are savings to Medicare in stringent CON states. Moreover, the Sloan and Conover analysis was performed using data through 1998, hence should have picked up any beneficial effects of CON on CABG spending. The only way to reconcile these results is to assume that Medicare and CABG savings are offset by corresponding increases in health spending elsewhere, resulting in zero overall net measured impact. Accordingly, the expected value was derived using an exponential distribution in @RISK so that the cost increase matched the combined savings reductions due to fewer CABG facilities and Medicare spending in stringent CON states.

[q] All figures are calculated by authors using Pennsylvania figures reported by Kolstad, who estimates the net addition of 10–16 CABG programs as a consequence of CON removal ([39] , p.34); the average annual number of CABGs is computed using figures on total number of CABG programs and average annual CABG surgeries, reported for the years 2000–2003 in Table 1.1 from the same source.

[r] All figures are calculated by authors using the same range reported by Kolstad ($10–12 million) ([39] , p.34), and adjusting these figures based on changes in the Turner Building Cost Index [43]. The $10 million figure is from 2001 [42] and the $14 million figure is an average from 1996 to 2000 data [41], so 1998 was assumed to be the base year for inflation adjustment purposes.

[s] All figures are calculated by authors using Excel’s PMT formula, where interest rate = 5% (the prevailing bank prime loan rate in July 2008 [44]), period = 15 years (as reported in [53]) and present value = capital cost per CABG program [P18].

[t] The expected value figure is reported in ([53] , p.28) and is based on the median hourly wage in Pennsylvania and the study’s finding that average patient travel distance was reduced by 2.3 miles due to the entry of new CABG facilities in the 5 years following elimination of CON in Pennsylvania. In May 2014, the median wage in Pennsylvania ($17.13) was nearly identical to the national average ($17.09) [47]. However, due to uncertainty about the extent that average patient distance would be increased in other CON states, the lower and upperr bounds were set at 50% below and 50% above the expected value.

[u] Figures are calculated by authors using total CABGs reported for 2008 [64] and allocating them to states based on their share of the total population age 18 and older, using Census estimates reported at [40]. Lower and upper bounds were imputed by authors using lower and upper bounds reported at original source of CABG data.

[v] Figures are reported in [49]. Lower and upper bounds represent 90% confidence interval using reported standard error of 1,462,000.

[w] The all-condition in-hospital rate of death for those aged 65 and over derived by author from HCUP Nationwide Inpatient Sample (NIS), 2008 tabulations created April 24, 2014 at [48]. Lower and upper bounds represent 90% confidence interval using estimated standard error of .06% derived by author using formula and parameters provided at source.

[x] Because it measured effects using data roughly 25 years old and also has its own methodological limitations, the Shortell and Hughes finding that mortality was 6% higher than expected in states with stringent CON (Table 4 at [16]) is used as the top 1 percentile cost estimate. For consistency with how other mortality data of that vintage have been treated, a weight of 25% is applied to this figure. The expected, lower bound and upper bound figures are calculated in @RISK assuming an exponential distribution with 1.5% as the 99% value.

[y] Figure calculated in [50].

[z] Figure shown is a weighed average from three studies calculated in Appendix Table A2. Lower and upper bounds have been calculated in @RISK.

[aa] Expected value figure is cited in [53]. To account for uncertainty in the gains in average life expectancy that may have occurred for the typical CABG patient by 2008 and because the actual average would depend on the age mix of CABG patients in CON states, the lower-bound figure was set 25% below expected; upper-bound figure = 25% above expected.

[ab] Expected value figure is for CABG patients alive after 10 years, from [54]. No standard errors are reported at the original source, hence lower and upper bounds are set at 25% below expected; upper-bound figure = 25% above expected.

[ac] Figure calculated in [50].

[ad] Figures are calculated by authors using total CABGs reported for 2008 [62] and allocating them to states based on their share of the total population age 18 and older, using Census estimates reported at [40] and states reported in [63] as having stringent CON in 2008. Lower and upper bounds were imputed by authors using lower and upper bounds reported at original source of CABG data.

[ae] Figure shown is calculated in Appendix Table A2 based on a single study. Lower and upper bounds have been calculated in @RISK.

[af] The expected value figure is calculated by authors using figures reported in Table 8 at [56] on lifetime gain in QALYs and number of patients for 3 subgroups: age < 60, age 60–69 and age 70 and older. The figure shown is biased upwards since it is based on quality-adjusted life expectancy of patients age 60 and older compared to the average across all ages.

[ag] The expected value figure is calculated by authors using total CABGs reported for 2008 [64] and allocating them to states based on their share of the population age 65 and older, using Census estimates reported at [40]. Lower and upper bounds were imputed by authors using lower and upper bounds reported at original source of CABG data.

[ah] Figure shown is calculated in Appendix Table A2 based on four studies. Lower and upper bounds have been calculated in @RISK.

[ai] This figure has been calculated using the same logic described in footnote E regarding health spending. The only study to explicitly assess the impact of stringent CON on CABG mortality is Popescu et al. [53], using 2000–2003 data, showing a decline of 7.31 deaths per 1000 elderly CABG patients in stringent CON states. But the same study showed no impact of CON on elderly CABG mortality rates, implying that lower rates in stringent CON states were counterbalanced by higher mortality experience in states with moderate or limited CON. The expected value figure is calculated by authors so that the estimated mortality gains in states with stringent CON are consistent with the weighted average impact of CON on mortality across all CON states: (Elderly CABGs in States with CON [P33]) x (Change in elderly CABG Mortality Rate/1000, All CON States [P34]) x - (Elderly CABGs in States with Stringent CON, 2008 [P30]) x (Change in Elderly CABBG Mortality Rate/1000 Due to Stringent CON [P31])/[(Elderly CABGs in States with CON [P33]) - (Elderly CABGs in States with Stringent CON [P30])].

[aj] Marginal overhead costs equal the sum of collections costs, compliance costs and marginal excess burden. Marginal excess burden is the efficiency loss associated with a small increase in taxes. It represents the share of the revenues collected that are lost due to reduced output as measured by general equilibrium models. The figures shown are weighted average marginal overhead costs as a percentage of federal tax revenues using the best available estimates from the literature for each, as reported in [60].

[ak] The figures shown are weighted average marginal overhead costs as a percentage of state tax revenues using the best available estimates from the literature for each, as reported in [60].

[al] Marginal excess burden is the efficiency loss associated with a small increase in sales taxes. It represents the share of the revenues collected that are lost due to reduced output as measured by general equilibrium models. Health industry compliance burdens may be viewed as roughly equivalent to an excise tax on industry sales. Therefore, the figures shown are marginal excess burden figures for general sales using the best available estimates from the literature for each. All figures are calculated in [60].

**Table 3:** Summary of Studies on the Effect of CON on Mortality for Coronary Artery Bypass Graft (CABG) Procedures

STUDY	DESIGN	STATES	YEARS	POP-ULATION	CONTROLS	ESTIMATED EFFECT OF CON ON MORTALITY						NOTES
						Mortality measure	Mean change^1^	Stand-ard error	Net change, deaths per 1000 patients^2^	Pop-ulation weight^3^	Recency weight^4^	
**Total Population Studies**												
Ho [65]	Retro-spective cohort	18 CON vs. 8 no CON	1988–2000	All	State fixed effects, patient characteristics	Inpatient mortality	−2.6%	0.8%	−1.00	77.1%	50.0%	Only one state in sample dropped CON during the study period. Finds no mortality effect of CON on PTCA. SE imputed from reported t statistic.
DiSesa et al. [52]	Retro-spective cohort	27 CON vs. 24 no CON	2000–2003	All	State and hospital fixed effects, patient controls	Operative mortality	−4.9%	5.7%	−1.25	100.0%	100.0%	
Robinson et al. [42]	Pre-post	PA	1994–1999	All	Patient characteristics	Inpatient mortality	0.0%	0.9%	0.00	0.0%	50.0%	After CON lifted, actual mortality matched expected mortality for both old and new cardiac programs; uses same PHC4 data as Kolstad
Kolstad [39]	Pre-post	PA	1994–2003	All	Compares incumbent hospitals to new entrants		2.9%	0.9%	0.62	4.3%	100.0%	Kolstad calculates that 11 deaths are averted annually by CON repeal. His Table 1.1 shows that in 2000–2003, the 40 incumbent hospitals performed an average of 349 CABGs, for a total of 13,960 (RAMR = 2.17%) while 24 new entrants performed an average of 160 (RAMR = 2.04%). Weighted average mortality = 2.14% vs. 2.20% if 11 additional deaths had occurred.
Cutler et al. [53]	Pre-post	PA	1994–2003	All	Compares incumbent hospitals to new entrants		2.9%	0.9%	0.62	0.0%	100.0%	Essentially the same paper as Kolstad [39]
Weighted average:									−1.13			
**Medicare Patient Studies**												
Vaughan-Sarrazin et al. [57]	Retro-spective cohort	27 continuous CON vs. 18 no CON	1994–1999	Medicare (excludes managed care)	Patient characteristics	In-hospital mortality	−17.3%	2.6%	− 8.70	77.1%	25.0%	States without CON exhibited CABG higher mortality (OR = 1.22) than states with continuous CON; this implies CON is associated with an 17.3% decrease in mortality rates, derived algebraically. No effect in intermittent CON states
Popescu et al. [58]	Retro-spective cohort	27 CON vs. 23 no CON	1998–2000	Medicare	Patient characteristics	30-day all-cause mortality	−5.0%	1.0%	−8.90	100.0%	50.0%	
DiSesa et al. [52]	Retro-spective cohort	27 CON vs. 24 no CON	2001	Medicare patients age 65 and older (excludes managed care)	State and hospital fixed effects, patient controls	Operative mortality	−0.3%	4.9%	−0.10	100.0%	75.0%	
Popescu, Vaughan-Sarazin and Rosenthal [55]	Retro-spective cohort	27 CON vs. 24 no CON	2000–2003	Medicare (age 68+)	Patient characteristics	30-day all-cause mortality	0.0%	1.5%	0.00	100.0%	100.0%	Vaughan-Sarazin is co-author on this paper; her most recent work, using the most recent data she uses, finds zero effect (a true 0.00 estimate; not just statistically insignificant)
Ho et al. [59]	Retro-spective cohort	27 continuous CON vs. 7 dropped CON	1989–2002	Medicare patients age 65 and older (excludes managed care)	State fixed effects, extensive hospital and patient controls	Procedural mortality	10.8%	3.3%	5.20	63.9%	100.0%	Dropping CON reduces mortality at first, but the effect dissipates 5 years after CON is removed
Weighted average:									−0.93			
Popescu, Vaughan-Sarazin and Rosenthal [55]	Retro-spective cohort	27 CON vs. 24 no CON	2000–2003	Medicare (age 68+)	Patient characteristics	30-day all-cause mortality	−4.2%	2.6%	−7.31	100.0%	100.0%	States with stringent CON lower mortality but effect is of borderline statistical significance

^1^Mean change in probability of death, calculated by authors using data reported at original source

^2^All figures calculated by authors: 1000 x (Mean Mortality Rate in CON States) x (1–1/(1 + Mean Change)) using data on the mean mortality rate for the relevant population and mortality measure shown as reported at original source

^3^Population weights represent the fraction of the theoretical population of interest included in a study. All figures are calculated by authors based on the total number of CABG surgeries in 2008, allocated to states based on 2008 Census figures on total adult population age 18 and older (for total population studies) and total population age 65 and older (for Medicare patient studies). A weight of zero has been assigned to studies that either duplicate other reported findings or have been entirely superseded by analyses using the same (overlapping) data source but with additional newer years of data

^4^Recency weights are calculated to provide greater weight to results that rely on more recent data and/or improved methods

**Table 4:** Summary of Studies on the Effect of CON on Quality for Procedures Other than Cardiac Surgery

STUDY	DESIGN	STATES	YEARS	CONTROLS	KEY FINDINGS	NOTES
Zinn [29]	Cross-sectional	50	1987	Facility characteristics	Moratorium on building makes nursing home patients 7% more likely to be physically restrained, reduced RNs per patient 32%	Physical restraint increases 2.5 pp. from a 36% base; RNs per patient falls 1.3 pp. from a .04 base
Ford and Kasserman [17]	Time series of cross sections	50	1982–1989	State characteristics	Both presence of CON and CON stringency have a significant negative impact on both entry of new firms and expansion of capacity in dialysis industry	No direct evidence on quality; authors show CON increases market concentration, then cite other work saying that concentration reduces quality

**Table 5:** Summary of Studies on the Effect of CON on Access

STUDY	DESIGN	STATES	YEARS	KEY FINDINGS
**National Studies**				
Fric-Shamji and Shamji [66]	Retrospective cohort	26	2004–2005	CON has 0% effect on procedure rates, but may shift care to non-profit hospitals
Fric-Shamji and Shamji [67]	Retrospective cohort	26	2004–2005	CON has 0% effect on procedure rates
Fric-Shamji and Shamji [68]	Retrospective cohort	26	2004–2006	CON has 0% effect on procedure rates, but may shift care to teaching hospitals
Popescu [55]	Retrospective cohort	50	2000–2003	CON reduces that chance that a patient with AMI is admitted for revascularization by 18%
Ho [65]	Retrospective cohort	50	1989–2002	CON results in 19.2% fewer PCIs being performed
Ho et al. [59]	Retrospective cohort	50	1989–2002	Removing CON increases PCIs and CABGs by 0%
Short et al. [69]	Retrospective cohort	50	1989–2002	CON has 0% effect on cancer resection procedures
Ho, Ross et al. [65]	Retrospective cohort	50	1989–2002	CON increases CABGs by 0%
**Case Studies**				
DeLia et al. [70]	Retrospective cohort	NJ	1995–2004	Removing CON decreases racial disparity in cardiac angiography by 3%
Robinson et al. [42]	Retrospective cohort	PA	1994–1999	Removing CON increases CABGs by 0%
Kolstad [39]	Retrospective cohort	PA	1994–2003	Removing CON decreases travel distance for CABG by 2.3 miles (9%)

**Table 6:** Summary of studies on effect of CON on health spending

STUDY	YEARS	CONTROLS	KEY FINDINGS
Conover and Sloan [2]	HCFA; 1980–1998	State characteristics, State fixed effects	Dropping CON has a 0% effect on all expenditures
Lanning, Morrisey and Ohsfeldt [13]	HCFA; 1969, 1972, 1976–1982	2SLS accounts for endogeneity of CON	CON increases hospital spending 20.6%, overall spending 13.6%
Hellinger [63]	No source reported; 1985, 1990, 1995, 2000	State characteristics	CON decreases hospital beds by 10%, which in turn decreases spending by 1.8%
Rivers et al. [71]	AHA; 1999–2003	Hospital and state characteristics, state fixed effects	CON has a 0% effect on hospital spending; strict CON increases hospital spending 4.9%
Grabowski [72]	CMS; 1981–1998	State fixed effects	CON repeal increases Medicaid nursing home expenditures 0%

## Appendix B

### Search strategies

**Table:**

**Database: Ovid MEDLINE(R) < 1975–2010>** Search Strategy #1: Costs.		
**#**	**Searches**	**Results**
1	exp certificate of need/	801
2	“certificate of need”.mp.	886
3	1 or 2	886
4	(cost$ or burden$ or impact$).mp.	736,742
5	exp “costs and cost analysis”/	157,748
6	ec.fs.	287,061
7	4 or 5 or 6	885,552
8	3 and 7	368
9	..l/ 8 lg = en	366
10	..l/ 9 yr = 1975–2004	325
11	..l/ 9 yr = 2005–2010	40

**Table:**

**Database: Ovid MEDLINE(R) < 1975–2010>** Search Strategy #1: Benefits.		
**#**	**Searches**	**Results**
1	exp certificate of need/	801
2	“certificate of need”.mp.	886
3	1 or 2	886
4	(mortalit$ or morbid$).mp.	489,833
5	exp Mortality/	235,682
6	exp morbidity/	298,906
7	4 or 5 or 6	855,189
8	3 and 7	18
9	access$.mp.	243,710
10	3 and 9	58
11	8 or 10	73
12	..l/ 11 lg = en	73
13	..l/ 12 yr = 1975–2004	49
14	..l/ 12 yr = 2005–2010	24

## Appendix C

### Detailed Literature Summaries

#### Hospital CON

- Using HCFA time series cross section state level per capita spending data for 1969, 1972 and 1976–1982, Lanning, Morrisey and Ohsfeldt [13] found that CON was associated both with an increase in per capita hospital spending (20.6%) and per capita spending on other health services (9.0%), for a net increase of 13.6% in health spending overall. They conclude that “there seems to be little economic justification for continuing CON regulation”. ([13] , p.174)
- Using the same HCFA data from 1980 to 1993, Conover and Sloan [12] found that CON had a long run effect of reducing hospital spending per capita by 5%, but there was no significant impact on overall health spending per capita.
- In an update that makes use of the same data updated through 1998, Sloan and Conover [45] find that dropping CON for acute care services had no significant effect on total per capita health spending, its components (hospital and physician spending) or Medicare spending per eligible. Stringent CON was associated with a statistically significant reduction in hospital spending, but not in overall per capita expenditures, along with a − 1.8% decline in Medicare spending per eligible.
- In a 2004 report, the Federal Trade Commission and U.S. Department of Justice, after extensively reviewing the available literature and hearing from expert witnesses, concluded the following: “The Agencies believe that CON programs are generally not successful in containing health care costs and that they can pose anticompetitive risks. As noted above, CON programs risk entrenching oligopolists and eroding consumer welfare. The aim of controlling costs is laudable, but there appear to be other, more effective means of achieving this goal that do not pose anticompetitive risks”. ([15] , p.5)
- Hellinger [63] used general estimating equations to examine the impact of CON on state-level per capita health expenditures in 4 years: 1985, 1990, 1995, 2000. Oddly, this study does not report the data source used for health expenditures, although it almost certainly made use of periodically issued estimates of state health expenditures that are released by Centers for Medicare and Medicaid Services. It found that states with certificate-of-need programs that cover short-term hospitals experience healthcare expenditures per capita that are 1.8% (41 × 0.045%) lower than those in states without such a CON program. Similarly, this implies that states with stringent certificate-of-need programs experience healthcare expenditures per capita that are 3.4% (76 × 0.045%) lower than those in states without stringent CON programs. While the analysis controls for many state characteristics, it does not use state fixed effects. Moreover, this core finding is undercut to some extent insofar as when CON is included as a covariate to explain health spending, it is not significant. The premise is that CON first reduces beds and this reduction in beds ultimately produces a later reduction in health spending. But it is puzzling that such a reduction in spending is not observed in the very same year that there are fewer beds etc.
- Ho, Ku-Goto, and Jollis [59], using Medicare inpatient claims data for 1989–2002, found that dropping CON does not appear to influence the statewide number of CABG or PCI procedures, but spreads these revascularizations over a larger number of facilities. Both CABG and PCI have significant fixed costs, and lower hospital volume has been associated with higher costs per patient for both of these procedures. Thus, it is possible this results in higher spending in states that dropped CON even though this was not measured directly. This study relied on a differences-in-differences model with state fixed effects to address endogeneity concerns.
- Rivers, Fottler, and Frimpong, [71] using a panel representing 2168 short-term general, nonfederal US hospitals operating during the period 1999–2003, show that CON has no statistically significant impact on cost per adjusted admissions for all hospitals. However, states with stringent CON regulations had higher costs per adjusted admissions for all hospitals. This replicates findings from numerous studies in the 1970s and 1980s but is noted here because the analysis used superior methods and examined a period after the introduction of the Medicare PPS for hospitals (1983) and widespread emergence of managed care in the 1990s. Researchers used a fixed-effect model relying on first-differences to overcome the limitations of prior studies.

**CON and Hospital Efficiency**. Several studies have examined CON’s impact on the measured efficiency of the hospital industry.

- In one of the earliest such studies, Eakin [73] estimated minimum costs based on a cross-section data of 331 U.S. short-term hospitals. To identify major contributors to inefficiency, hospital-specific estimates of allocative inefficiency were regressed against hospital attributes and regulation variables. Estimated coefficients on the binary variable of CON were positive and significant, showing that hospitals subject to CON regulations have a greater level of inefficiency by .88 to 1.03%.
- Bates, Mukherjee, & Santerre [74] measured technical efficiency using a technique known as data envelopment analysis (DEA). They then regressed the efficiency scores on a dummy variable for CON regulation and other elements of market structure (e.g., HMO activity). Based on their regression results, the authors concluded that, on average, CON laws did not reduce efficiency.
- Using data from 1994 to 2002, Ferrier, Leleu, and Valdmanis [75] found that in general, the hospital sector in states with active CON regulations had 2.1% lower aggregate technical inefficiency, 0.8% lower structural inefficiency and 2.0% lower mix inefficiency, irrespective of the stringency or laxness of this oversight. “Hence, the presence of any CON has a positive impact measured by improved resource allocation and by definition, lower social costs.” However, these efficiency gains were counterbalanced by 1.3% higher scale inefficiency, implying that hospitals in CON states tended to have more excess capacity even though CON in principle was designed to curtail this. It is worth noting that this model contained time fixed effects, but not state level fixed effects, controlling only for the percent of population living in rural areas and the share of hospital beds in private, public and federal hospitals. Without controlling for endogeneity of CON adoption, it is conceivable that other unmeasured differences between the states are driving this result.

#### Nursing Home CON

- Using a random effects model on data for 1991 through 1997 with states as the unit of analysis, Miller, Harrington, Ramsland, and Goldstein [76] found that the presence of either a nursing home CON, a nursing home moratorium or both a CON and moratorium had a (positive) statistically insignificant effect on nursing home care per capita expenditures. The same study did find that the presence of a nursing home CON, a nursing home moratorium and both a CON and moratorium had a positive and significant effect on Medicaid long-term per capita expenditures. However, we did not include that result in this analysis on grounds that the first result suggests that any Medicaid savings may have been offset by increased nursing home spending by other payers. Additionally, this study did not control for state or year fixed effects.
- In contrast, Grabowski, Ohsfeldt, and Morrisey [72] used a fixed effects model on data for 1981 through 1998, to account for unobserved state-specific and time-specific factors that could have influenced either the elimination of nursing home CON or moratoria or the level of nursing home and long-term care expenditures. His study showed that states without CON or moratorium policies have a (very small positive) statistically insignificant effect on both Medicaid nursing home spending as well as Medicaid long-term care outlays.
- Chi-Chang ([77] , p.101) shows that nursing home CON or moratoria are associated with a 1–3 percentage point decrease in cost inefficiency but a 1.4 percentage point reduction in technical efficiency ([78] , p.102).

#### Other Types of CON

- **Home Health CON.** Anderson and Kass [46] using 1981 Medicare cost reports for 1764 home health agencies (HHAs), found no evidence that CON regulation resulted in economies of scale or scope. That is, there was no significant difference in the extent of unrealized economies of scale or the extent to which nursing home and home health were jointly provided in states with CON regulation compared to states without it. At the individual firm level, the weighted average cost per visit was 2% higher in states with CON (*p* = .10). But this average masked important differences by ownership: non-profit HHAs had 4% higher costs in CON-regulated states (*p* = .01) but government-owned HHAs had costs that were 5.7% lower (*p* = .05) and the cost difference for for-profit firms was not statistically significant ([46], pp.88-90). The authors also performed a market-wide analysis that in one model found that costs were higher in CON states whereas a different model showed no significant difference.
- There are four important limitations of these findings. First, they are based only on Medicare-certified agencies rather than the entire home health market. Second, the study is entirely cross-sectional, with no effort to use state fixed effects or instrumental variables to control for endogeneity. Third, they are based on data more than a quarter-century old; this is especially problematic because Medicare shifted to a prospective payment system for home health in October 2000, [78] which may have completely altered the extent to which CON regulation might influence home health spending. Finally, the market-wide cost regressions produced estimates of marginal costs for selected types of home health services that in some cases were negative, which obviously is logically impossible. The authors conclude: “the results presented here should probably only be read as providing rough, impressionistic evidence of the effects of CON regulation of home health care market. The estimated regressions may not in general represent a reasonable aggregate cost function. However, these results are certainly consistent with the earlier evidence that CON does not result in lower costs” ([46] , p.107). For all these reasons, none of these findings were considered sufficiently reliable to include in the net assessment.
- **Hospice CON**. Carlson [79] used data from 1992 to 2000 to show that states that did not have CON regulation of hospices experienced a statistically significant increase in the number of hospice entrants during this time period; market areas (defined as MSAs or counties in areas not part of MSAs) without CON experienced .72 more hospice entrants over the 9 year period. Since hospices are associated with savings of $2309 per user [80] and two-thirds of hospices serve under 100 users annually ([79] , p.34) each hospice foregone in a market area represents $230,000 in potential annual savings lost. Based on OMB [81] data, a typical state with hospice CON regulation would have 46 market areas; this would imply potential annual savings foregone of $850,000 per state.
- **Kidney Dialysis**. Ford and Kaserman [17] analyzed the impact of CON regulations on entry into the dialysis industry from 1980 to 1989 showing that these regulations have constrained entry and expansion in this industry, culminating in higher costs for dialysis.

***Beneficial Effects of CON on Mortality***

Three studies have found that CON is associated with a reduction in mortality.

**Coronary Artery Bypass Surgery (CABG)**. Two national studies have shown a beneficial effect of CON on mortality following CABG surgery.

- Vaughan-Sarrazin Hanna, Gormley, and Rosenthal [57] conducted a national retrospective cohort study using Medicare Provider and Analysis Review (MedPAR) Part A data for over 900,000 patients admitted to over 1000 hospitals across all 50 states from 1994 through 1999. Risk-adjusted mortality was 22% higher in 18 states that had no CON regulation of open heart units during this period compared to 26 states that maintained such regulation during the entire period. Mean patient volume per center in the 26 states with continuous CON was 84% higher than in the 18 states with no CON (191 annual cases vs. 104). Thus, a significantly higher percentage of cases were performed in low-volume hospitals, consistent with the hypothesis that CON led to improvements in outcomes by regionalizing facilities. While this study controlled extensively for various patient characteristics, it did not control for other factors such as Medicare managed care penetration, physician-to-population density, or physician specialty mix, regional differences in the use of PTCA or various efforts by states to report CABG outcomes to consumers. The exclusion of patients in Medicare managed care plans may be particularly important: “therefore, the differences in hospital volume and mortality identified in this study may be upward biased due the higher rate of managed care penetration in states without CON regulation in their sample” [51]. Moreover, its cross-sectional nature limits the ability of the authors to draw cause-and-effect conclusions, as it is conceivable that states without CON regulation of open heart surgery had worse surgical outcomes anyway for reasons unrelated to CON. More concretely, it does not include state fixed effects or other methods to address endogeneity. Consequently, much (or all) of the large mortality differential reported may be due to factors which are related to the presence or stringency of CON regulation in a state.
- Ho [51] using AHRQ HCUP National Inpatient Sample data from 1988 to 2000, found that CON regulations increase patient volume (14 years after dropping CON, the average number of CABG procedures per hospital declined by 27%). Using the measured relationship between procedure volume and risk-adjusted inpatient mortality, the author calculated that “for an average hospital in a state 14 years beyond repeal of CON rules, inpatient mortality rates for CABG are .1 percentage points higher than they otherwise would have been” ([51] , p.315). This compares to an average risk-adjusted mortality rate in states that dropped CON of 3.9% in 2000. Further calculations by Ho showed that the average state dropping CON would experience roughly 4 additional CABG deaths a year due to this decline in patient volume. A limitation of this study is that all of the states in the HCUP sample except one discontinued their cardiac CON program prior to 1988. Therefore, the analysis focused on examining differences in hospital procedure volume as a function of the number of years since CON legislation was repealed in a given state. Another shortcoming is that the study mixes mortality changes for states that dropped CON in the mid-1980s with those of states that dropped cardiac CON regulations more recently. This may yield misleading results if technology for cardiac surgery has improved over time, since combining the mortality changes following CON removal from two distinct periods along technology continuum could mask the magnitude of potential mortality changes currently available to states dropping CON.
  **Coronary Revascularization (CABG or PTCA).** In a retrospective cohort study of nearly 800,000 Medicare beneficiaries with AMI who were admitted to 4587 US hospitals during 1998–2000, [58] found that compared to patients in states without CON, patients in states with CON regulations had 6% lower 30-day mortality risk. It is worth noting that a follow-up study using the same methods but more recent data from 2000 to 2003 found no mortality differences in CON vs. non CON states [55]. As well, this study design has important weaknesses detailed in the discussion of the latter study results.

***No Effects of CON on Mortality***

Five studies have found no statistically significant impact of CON on mortality risk.

**Coronary Artery Bypass Surgery (CABG)**. Two studies have found no effect of CON on mortality following CABG surgery.

- **National Study of Cardiac Patients**. Using cardiac registry data from 2000 to 2003, DiSesa et al. [52] show that CON states have significantly higher hospital average CABG surgery volumes but similar mortality compared with non-CON states. Thus, even though CON evidently succeeded to some extent in regionalizing CABG surgeries, this did not have the intended effect of reducing mortality. This study used more detailed clinical data and controls for regional confounding than Vaughan-Sarrazin et al. [57] although it too does not include state fixed effects. The study also included subanalyses using state random effects: these account for some unobserved heterogeneity, but not all types.
- **Pennsylvania Case Study**. Robinson, Nash, Moxey, and O’Connor [42] used inpatient data on all CABG surgeries performed in Pennsylvania hospitals between 1994 and 1999 to compared CABG outcomes 3 years prior to Pennsylvania’s elimination of CON to 3 years afterwards. Despite a 25% increase in the number of open-heart surgery programs once CON was lifted, the authors found no significant difference in the mortality experience of hospitals that were approved under CON to perform CABG compared to those that developed open-heart programs following CON’s removal. Thus, the advantage of this study is that it is a longitudinal comparison that focuses on the impact of lifting CON. The disadvantage is that it is a case study of a single state, and one that happened to also have a statewide public performance monitoring system that included hospital reporting of CABG outcomes. Absent this reporting system, it is conceivable that very different results might have been found.

**Coronary Angioplasty (PTCA: Percutaneous Transluminal Coronary Angioplasty)**. Two studies have found no effect of CON on mortality following PTCA procedures.

- **National Study of Cardiac Patients**. Ho [51] using AHRQ National Inpatient Sample data from 1988 to 2000, found that although CON appears to lead to a sizeable increase in average hospital PTCA volume, there is no evidence that beneficial reduction in inpatient mortality accompanies this volume change.
- **California vs. Florida Case Study**. Ho [18] using hospital discharge data for 1988–1998, found that Florida CON laws were associated with higher average PTCA volumes per hospital relative to California hospitals, where no such laws exist. Simulations showed that increasing the mean PTCA volume in California from its 1998 level of 389 procedures to the Florida mean of 724 would not change inpatient mortality rates, although the probability of urgent bypass grafting surgery would decline from 2.2 to 2.0% for a representative patient. The author concluded that “because a higher PTCA volume was associated with moderately better outcomes, CON may be marginally effective in improving outcomes for PTCA.”

**Coronary Revascularization (CABG or PTCA).** In a retrospective cohort study of 1.1 million Medicare beneficiaries aged 68 years or older with AMI who were admitted to 4587 US hospitals during 2000–2003, Popescu et al. [55] found that the odds of 30-day mortality were similar in states with CON relative to states without CON. Adjusted odds of death within CON stringency groups were of borderline significance for high stringency certificate of need states (OR, 0.95; 95% CI, 0.90–1.00; *P* = .07) but were similar for moderate- and low stringency CON states. The authors speculate that “higher hospital volumes in states with certificates of need may counteract any adverse effects of limiting access to revascularization services” ([55] , p.2146). The authors concede “the study design cannot discern a cause-and-effect relationship between certificates of need and use of revascularization or mortality” [55]. The analysis did not control for a variety of factors that might have contributed to observed mortality differences including managed care penetration, regional physician practice variation, concurrent efforts to improve quality, or differences in other diagnostic and therapeutic choices (e.g., use of thrombolytics, aspirin, beta-blockers), as these were not captured by administrative data.

***Adverse Effects of CON on Mortality***

There is only one study that has shown adverse effects of CON on mortality in general. All of the remaining studies have focused on CON’s adverse impact on CABG outcomes, one done with national data and the remainder using single state case studies.

**Medicare Mortality Risk**. Using Medicare inpatient claims data from 1983 to 1984 for 981 of the nation’s 6500 hospitals located in 45 states, Shortell and Hughes [15] examined condition-specific mortality rates among hospital inpatients for 16 selected diagnoses, showing that the ratio of actual to expected deaths was 5–6% higher in states with stringent CON regulation compared to states with less stringent CON. This study was done before any states had dropped CON, so the only available comparison was between more restrictive and less restrictive CON programs. The ratio of actual to expected deaths was 1.06 in stringent CON states, .966 in states with medium stringency and .999 in states with the least stringent CON regulations. The authors conduct a statistical test showing the association of CON stringency with mortality is statistically significant, but do not provide pairwise comparisons of medium stringency vs. low CON states, so there is no way of knowing whether this difference is statistically significant. These hospitals were not randomly selected and there is a fierce debate over the credibility of these results; accordingly, we use them only as an upper bound.

**National CABG Studies**. Only one of the CABG studies used national data. Ho et al. [59] using Medicare inpatient claims data for 1989–2002, found that states that dropped CON experienced lower CABG mortality rates relative to states that kept CON, although the differential is not permanent. This analysis examined procedural mortality for CABG or PCI (death during the same hospitalization as revascularization, or after discharge but within 30 days of surgery) and relied on a differences-in-differences model with state fixed effects to address endogeneity and unmeasured variable concerns.

**CABG Case Studies**. Because of the intrinsic uncertainty about how accurately their findings can be generalized to other states, case study evidence generally is weaker than studies examining many or all states.

- Kolstad [39] found that repeal of CON in Pennsylvania led to a redistribution of CABG surgeries from lower- to higher-quality surgeons. Specifically, as new programs entered the market, volume shifted from incumbents to new entrants and from lower- to higher-quality surgeons. He estimates that the value of the improved outcomes resulting from this shift—11 deaths averted annually, or 88 life years--was roughly equal to the additional fixed costs incurred by new entrants ($13 million per program). Thus, overall social welfare neither increased nor decreased from removal of CON as it relates to CABG surgery. This is the first CON study to explicitly assign an economic value to mortality gains resulting from CON removal.
- A peer-reviewed version of Kolstad’s findings is reported in Cutler, Huckman, and Kolstad [51].

#### Other Health Outcomes

- **Kidney Dialysis**. Ford and Kaserman [17] analyzed impacts of CON regulations on entry into the dialysis industry from 1980 to 1989 showing that these regulations have constrained the entry and expansion in this industry; which led to a decrease in the quality of care for dialysis.
- **Coronary Angioplasty (PTCA: Percutaneous Transluminal Coronary Angioplasty)**. Ho [18] using hospital discharge data for 1988–1998, found that Florida CON laws were associated with higher average PTCA volumes per hospital relative to California hospitals, where no such laws exist. Simulations showed that increasing the mean PTCA volume in California from its 1998 level of 389 procedures to the Florida mean of 724 would reduce the probability of urgent bypass grafting surgery from 2.2 to 2.0% for a representative patient. The author concluded that “because a higher PTCA volume was associated with moderately better outcomes, CON may be marginally effective in improving outcomes for PTCA” ([18] , p.442). But the author also cautions: “lack of access to angioplasty as a result of regionalization may have negative health consequences for patients in less populated areas who require emergency angioplasty” ([18] , p.447).
- **Cardiac Catheterization**. Ross et al. [19] examined chart-abstracted data for more than 137,000 Medicare patients admitted for acute myocardial infarction between 1994 and 1996 at 4179 US acute-care hospitals. Patients were categorized into three groups: a) whether they had characteristics suggesting catheterization was generally recognized as “beneficial, useful, and effective” (strong indication); b) were patients for whom data on the effectiveness of the procedure were unclear (equivocal indication); or c) were patients who had conditions for which cardiac catheterization was considered unlikely to be effective (weak indication), Using multi-level modeling to account for a variety of patient, hospital and state characteristics, the authors found that CON regulation was associated with 12% lower rates of catheterization among patients with equivocal indications and a 16% lower rate of catheterization among patients with weak indications. There were no significant differences in catheterization rates among patients with strong indications. The authors conclude that this “suggests either that physicians do discriminate on the basis of procedure appropriateness when faced with reduced capacity to provide care or that facilities refer fewer less appropriate patients for catheterization when greater facility volume is ensured” ([19] , p.1016). The authors also caution: “we found substantial underuse of appropriate care, because only 50% of patients with strong indications in states with and without CON regulation received cardiac catheterization after admission for AMI.” Thus, CON may improve quality by reducing use of catheterization when not medically appropriate, but it does not appear to increase the use of catheterization when it is appropriate either. The authors further note that the observational, cross-sectional design can only suggest but not prove a cause-and-effect relationship between CON regulation and use of cardiac catheterization.

#### Positive Effects of CON on Access

Some research has shown that some states explicitly use their CON authority to protect existing providers that serve underserved populations [82, 83].

***Access to Disadvantaged Populations***

**National Studies**.

- Miller and Hutton (2000; cited in [32] , p.56) show that state health agencies gave favorable treatment in the CON review process to hospitals with a high volume of Medicaid and indigent patients.
- Using 2002–2004 State Inpatient Data HCUP data on hospitals in 12 states with CON and 5 states without CON, Zhang [84] assessed the impact of CON on the number of uninsured hospital admissions and percentage of hospital admissions due to uninsured patients. He controlled for a variety of market and hospital characteristics and used both a generalized least squares (GLS) model as well as an instrumental variable model designed to account for endogeneity. He found that CON laws increased both the number nonprofit hospital admissions for the uninsured (11 per year per hospital) and uninsured percent admissions (by 0.07%.). These small effects were significant in GLS model (which did not account for endogeneity); somewhat larger effects were found using the IV model but were not statistically significant. GLS results did not show that CON laws have any effect on for-profit hospital’s number of uncompensated care admissions; however, CON laws are significantly positively related to the percent of admissions for the uninsured by for-profit hospitals, again by a small amount (0.25%). The IV model produces much larger effects (6.9% increase) but these are not significant. Zhang concedes weaknesses in both models but emphasized the consistency in general direction of results from both as an indication that what was being measured was a genuine phenomenon.

**Case Studies.**

- **California**. Campbell and Ahern [82] used two-period California data to explore the effect of CON on uncompensated care provision. Specifically, they run separate multivariate regressions for California hospitals in 1963 and 1987 to examine the determinants of hospital provision of uncompensated care. They found a positive relationship between net profitability of private nonprofit hospitals and the amount of uncompensated care they provide. They argue that this finding suggests government regulators reward heavily burdened uncompensated care providers with profitable CON licenses. Since no CON variables are actually used in estimating the amount of uncompensated care given by providers, this study fails to demonstrate a direct connection between CON activities and actual provision of indigent care.
- **Florida**. Both Campbell and Fournier [83] in a descriptive study, and Fournier and Campbell [85] using an empirical model, have shown how Florida CON regulators used the licensing power to create entry barriers forcing existing providers to subsidize indigent care with higher than normal profit from other services. They found that, controlling for the endogeneity of indigent care, regulators in Florida systematically awarded CON licenses to hospitals providing greater amount of care to the poor. The authors noted that while this method of financing the indigent care may be preferred by legislators who do not want to face the political consequences of increasing taxes to pay for the service, the undesirable effects of restraining the provision of hospital services must be taken into account.
- **Ohio**. Mendelson and Arnold [86] (1993) found that regulators in Ohio used CON to protect access to care for the disadvantaged by denying applications that could have adverse effects on the financial viability of safety net hospitals in inner cities. Lewin and Alpha Center’s 1991 report (cited in [84] p. 17) to the Ohio Department of Health provided similar evidence.
- **Pennsylvania**. In Pennsylvania, the CON program also tended to reward providers who agreed to supply more uncompensated care [87].

***Access for General Population***

**Selected Surgical Procedures**. In a retrospective study that used HCUP State Inpatient data compiled by the Health Care Utilization Project, Fric-Shamji and Shamji [67] examined mean per capita rates of 6 surgical procedures in 21 states with CON laws and 5 states without between 2004 and 2005. While CON laws did not affect procedure rates, various procedures exhibited a shift from for-profit to nonprofit facilities including lumbar discectomy (20% vs. 9%), acoustic neuroma resection (5.5% vs.0.2%), MVD (20% vs.3%), and rotator cuff repair (23% vs. 10%). CON status had no effect on proportion of cases occurring at teaching facilities. Thus, CON regulation does not negatively affect resident access to sufficient caseload for surgical training. This study has a very weak design as it does not control in any way for differences between states that might have accounted for observed differences in surgery rates.

**Selected Surgical Procedures**. In a retrospective study that used HCUP State Inpatient data compiled by the Health Care Utilization Project, Fric-Shamji and Shamji [68] examined mean per capita rates of 26 diverse surgical procedures were evaluated in 21 states with CON laws and 5 states without between 2004 and 2006. Rates were higher in CON states for some procedures and lower for roughly the same number. However, when the comparison was limited to procedures performed in teaching hospitals, there were 12 procedures for which this rate was higher by a statistically significant amount in CON states and only 1 (cardiac transplant) in which CON states had a lower rate than states without CON. The authors conclude: “Increased procedural rates were seen in more profitable surgeries, such as cardiac and orthopedic, whose surpluses may then be used to fund medical care to the poor and uninsured. These results suggest that health care planning agencies may be strategically granting certificates to teaching facilities as a means of preserving uncompensated health care” [67, p.E83] This study has a very weak design as it does not control in any way for differences between states that might have accounted for observed differences in surgery rates.

#### No Effects of CON on Access

At least one study has found evidence that CON has no impact on access to selected surgical procedures.

- **Selected Surgical Procedures**. In a retrospective study that used HCUP State Inpatient data compiled by the Health Care Utilization Project, Fric-Shamji and Shamji [67] examined mean per capita rates of 6 surgical procedures were evaluated in 21 states with CON laws and 5 states without between 2004 and 2005. CON laws did not affect procedure rates for any of the surgeries examined (lumbar discectomy, carpal tunnel release, acoustic neuroma resection, microvascular decompression for trigeminal neuralgia, tonsillectomy and adenoidectomy, and rotator cuff repair). This study has a very weak design as it does not control in any way for differences between states that might have accounted for observed differences in surgery rates.
- **Selected Neurological Procedures**. An earlier abstract published by the same authors [66] using the same data sources and methods also reports no impact of CON on rates per 100,000 for lumbar discectomy. This appears to be a duplicative finding except that the abstract reports the comparison as being between 16 (rather than 21) states with CON versus 5 states without Thus, we report it for completeness but do not attach much weight to it given the likelihood it is duplicative and because the later version was peer-reviewed.

#### Negative Effects of CON on Access

***Access to Disadvantaged Populations***

The Lewin Group [36] conducted a comparison of 3 year rolling aggregate total margins between safety-net hospitals and non- safety-net hospitals in CON versus non-CON States using Medicare cost report data for the years 2003–2005. They found that even after controlling for geographic region, payer mix, proportion of uninsured population in the state, Herfindahl index for the hospital market concentration, occupancy rate, bed size and teaching status, safety-net hospitals in non-CON states had higher margins than safety-net hospitals in CON states (0.69% compared to − 0.02%). The authors concluded: “collectively, these results do not support the argument that CONs provide a protective effect for safety-net hospitals’ financial status ([36] , p.28). Similarly, in non- safety-net hospitals, the statistically adjusted margins in non-CON states were 4.0% vs. 1.91% in states with CON. The authors conclude: “Hospitals generally favor such laws in the belief that they restrict competition, allowing them to raise prices, but the results do not appear to support this belief.” This casts doubts on CON’s effectiveness in allowing hospitals to cross-subsidize care for uninsured or indigent patients.

***Access for General Population***

The Federal Trade Commission and U.S. Department of Justice [15] after extensively reviewing the available literature and hearing from expert witnesses, concluded the following: “The Agencies believe that CON programs are generally not successful in containing health care costs and that they can pose anticompetitive risks. As noted above, CON programs risk entrenching oligopolists and eroding consumer welfare. The aim of controlling costs is laudable, but there appear to be other, more effective means of achieving this goal that do not pose anticompetitive risks *A similar analysis applies to the use of CON programs to enhance health care quality and access*”.(emphasis added [14, Chapter 8, p. 5]).

A number of studies show that CON reduces the number of facilities offering a given regulated service yet no statistically significant change in the number of procedures. There is substantial empirical evidence that distance or travel time are important factors in patient choice of hospitals [88–91]. Accordingly, we have treated studies demonstrating a reduction in supply of facilities as adversely affecting access since fewer available facilities implies greater patient travel times for care.

Moreover, Feldstein ([92] cited in [32]) codifies many cases in which the courts found that the CON process was used in an arbitrary and capricious manner against new hospitals attempting to enter the market of an existing provider. Thus, CON regulation appears to create an equity issue even if there were no adverse effects on access to care.

**Coronary Revascularization (CABG or PTCA).** In a retrospective cohort study of 1.1 million Medicare beneficiaries aged 68 years or older with AMI who were admitted to 4587 US hospitals during 2000–2003, Popescu et al. [55] found that compared to patients in states without CON, patients in states with CON regulations were a) less likely to be admitted to hospitals with coronary revascularization services (51.5% vs 62.8%) b) less likely to undergo revascularization at the admitting hospital (26.1% vs 31.8%); and c) more likely to undergo revascularization at a transfer hospital (11.7% vs. 8.9%), all statistically significant results. But it is important to note these differences did not result in measured mortality differences in CON vs. no CON states. As well, the authors concede “the study design cannot discern a cause-and-effect relationship between certificates of need and use of revascularization or mortality” ([55] , p.2146). The analysis did not control for a variety of factors that might have contributed to observed mortality differences including managed care penetration, regional physician practice variation, concurrent efforts to improve quality, or differences in other diagnostic and therapeutic choices (e.g., use of thrombolytics, aspirin, beta-blockers), as these were not captured by administrative data.

**Percutaneous Coronary Intervention (PCI)**. Two national studies have produced mixed results, both showing that CON reduced the number of hospitals performing PCI but only one showing an actual reduction in the number of procedures.

- Using national data for 1989 and 2002, Ho, Ross, Nallamothu, and Krumholz [65] found that the presence of continuous CON regulations was associated with fewer hospitals per capita performing PCI. Multivariate regressions indicated that the presence of CON regulations was associated with 19.2% fewer PCIs per 1000 elderly in the population.
- Ho et al. [59] used the same 1989–2002 data but this time used a difference-in-difference-in-difference analysis with state fixed effects showing that removal of state cardiac CON regulations is associated with a 12.1% increase in the number of hospitals performing PCI. But there was no significant association between removal of CON and the total number of procedures performed.

**Coronary Artery Bypass Grafts (CABG).** Four national studies and two case studies have produced mixed results. Most of the evidence points to CON reducing the number of facilities that perform a given procedure without reducing the aggregate annual number of such procedures performed in that state; in other cases, CON has an adverse effect on population-adjusted procedure rates.

- Ho [51] using AHRQ HCUP National Inpatient Sample data from 1988 to 2000, found that CON regulations increase the mean number of CABG procedures per hospital. The author concludes: “These data are consistent with the hypothesis that CON restricts entry, allowing CON hospitals to grow larger on average.”
- This is consistent with the findings of Ho et al. [65]: using national data for 1989 and 2002, the authors found that each year, the per capita number of hospitals performing CABG was higher in states without CON (3.7 per 100,000 elderly for CABG in 2002), compared with CON states (2.5 for CABG in 2002). However, multivariate regressions found no difference in CABG utilization rates between states with and without CON.
- Similarly, using cardiac registry data from 2000 to 2003, DiSesa et al. [52] show that CON states have significantly higher hospital average CABG surgery volumes.
- Popescu et al. [55] found greater revascularization rates for patients with acute myocardial infarction (AMI) in non-CON versus CON states and indirect evidence of fewer facilities performing revascularization in CON states.
- Ho et al. [59] used Medicare inpatient claims data for 1989–2002 in a difference-in-difference-in-difference analysis with state fixed effects, showing that removal of state cardiac CON regulations is associated with a 15.2% increase in the number of hospitals performing CABG. However, there was no significant association between removal of CON and the total number of procedures performed.
- **Pennsylvania Case Studies**.
  - Robinson et al. [42] compared CABG outcomes 3 years prior to Pennsylvania’s elimination of CON to 3 years afterwards, showing a 25% increase in the number of open heart surgery programs once CON was lifted, but no significant increase in the number of CABG surgeries performed.
  - Kolstad [39] showed that in the 7 years following repeal of CON in Pennsylvania, the number of CABG programs grew by 49%. By comparing trends in New Jersey and New York during the same period, the author concluded that between 10 and 16 of the 24 new programs added could be attributed to CON repeal. The average CABG recipient traveled 2.3 fewer miles (a 9% reduction in travel distance); this amount was valued at $7.50 patient inclusive of family visits. Additionally, 15% of incumbent facilities accepted Medicaid patients compared to 10% of new entrants (although it also should be noted that most new entrants were located in suburbs).

**Cardiac Angiography**. Using N.J. data from 1995 to 2004, DeLia, Cantor, Tiedemann, and Huang [70] found that a CON reform designed to expand cardiac angiography (CA) capacity increased CA utilization overall and did so more rapidly for blacks, leading to a large reduction in the disparity. However, this reduction was *not* attributable to services provided by new entrants to the CA market, since they were located in mostly white suburban areas. Instead, the new entrants cut into the incumbents’ share of white CA patients who had previously traveled from the suburbs to receive the procedure at inner-city incumbent hospitals. As a result, it appears that incumbents were forced to serve more black patients in their local area to maintain their CA volume. These findings suggest that prior restrictions on CA capacity contributed to the historical disparity in access to the procedure.

**Carpal Tunnel Release**. Fric-Shamji and Shamji [66] compared rates of CTR and lumbar discectomy in 16 states with and 5 states without CON, finding CTR rates per 100,000 population were 22% lower in states with CON (in contrast, the difference in rates for lumbar discectomy was not statistically significant).

**Cancer Procedures**. Short, Aloia, and Ho [69] examined Medicare data from 1989 to 2002 for beneficiaries treated with one of six cancer resections and an associated cancer diagnosis. The study found that hospital availability was higher in non-CON than CON states, yet procedure use was similar across all states. Correspondingly, hospital procedure volume tended to be higher in CON states than in non-CON states. The number of hospitals per cancer incident was lower in CON states versus non-CON states for colectomy, rectal resection, and pulmonary lobectomy. Hospital volume was significantly higher in CON states versus non-CON states for colectomy and pulmonary lobectomy. There were no differences between states with and without CON in the number of procedures per cancer incident. This was a cross-sectional design that controlled for a number of state characteristics but not state fixed effects, so causal inferences are limited.

**Home Health**. Anderson and Kass, [46] using 1981 Medicare cost reports for 1764 home health agencies, found that in states with CON regulation of home health, there was an 11.6% reduction in the number of home health agencies in the average market, a difference that was statistically significant at the 5% level.

## Abbreviations

BLS: Bureau of Labor Statistics
CABG: Coronary Artery Bypass Grafting
CBO: Congressional Budget Office
CMS: Centers for Medicare and Medicaid Services
CON: Certificate of Need
HCFA: Health Care Financing Administration (superseded by CMS)
HMO: Health Maintenance Organization
HRET: Health Research and Education Trust
IPA: Individual Practice Association
OHS: Open heart surgery
OHU: Open heart unit
PTCA: Percutaneous Transluminal Coronary Angioplasty
QALY: Quality-adjusted life year, i.e., a year of life in perfect health
