# Supplementary material for: Certificate of need laws: a systematic review and cost-effectiveness analysis
Source: BMC Health Serv Res. 2020 Aug 14;20:748. doi: 10.1186/s12913-020-05563-1 (PMC7427974; doi:10.1186/s12913-020-05563-1)
Supplement: Supplementary file 1 — Additional file 1. [file 12913_2020_5563_MOESM1_ESM.docx]

# Listing of Excluded Studies

This list includes only studies that were excluded after full text review.

**Key for Reasons for Exclusion**

1. Studies with no original data

2. Studies with no outcomes of interest

3. Studies superseded by updated/published version

4. Studies published in abstract form only

American Health Planning Association. Certificate of Need coverage summary by state, 2010. Fairfax, VA: AHPA; 2011. Full text: exclude #2

Birkmeyer JD, Siewers AE, Marth NJ, Goodman DC. Regionalization of high-risk surgery and implications for patient travel times. Jama. 2003;290(20):2703-8. Full text: exclude #2.

Care Outcomes Assessment Program. 2010 risk-adjusted CABG mortality rate [Internet]. Seattle, WA: Foundation for Health Care Quality; 2014 [cited 2017 Nov 5]. Available from: <http://www.coap.org/2010-risk-adjusted-cabg-mortality-rate>. Full text: exclude #2.

Chen H. The Impact of BBA, HMOs, and hospital competition on quality of cardiac care [PhD [dissertation]]. Richmond, VA: Virginia Commonwealth University; 2008. Full text: exclude #2.

Gertler PJ. Medicaid and the Cost of Improving Access to Nursing Home Care. National Bureau of Economic Research Working Paper Series. 1989;2851. Full text: exclude #3

Guterman S. Specialty hospitals: a problem or a symptom? Health Aff (Millwood). 2006;25(1):95-105. Full text: exclude #2.

Hollingsworth JM, Ye Z, Strope SA, Krein SL, Hollenbeck AT, Hollenbeck BK. Physician-ownership of ambulatory surgery centers linked to higher volume of surgeries. Health Aff (Millwood). 2010;29(4):683-9. Full text: exclude #2.

Kahn CN, 3rd. Intolerable risk, irreparable harm: the legacy of physician-owned specialty hospitals. Health Aff (Millwood). 2006;25(1):130-3. Full text: exclude #2.

Kao D. State variations in linguistic competency policies and the effects on immigrant access to health services [PhD [dissertation]]. Los Angeles, CA: University of Southern California; 2010. Full text: exclude #2.

King AS. Medical market failure in Maine: Is the Dirigo Reform Act's Certificate of Need a market correction? Maine Bar Journal. 2007;22:156. Full text: exclude #2.

Markenson AJ, Rabbitt CK. Fifty state survey of Certificate of Need and licensure: Nursing homes, assisted living, home health, and hospice. Washington, DC: American Health Lawyers Association; 2009. Full text: exclude #2.

Masaki Y. The effects of cost-saving efforts in the U.S. healthcare market [PhD [dissertation]]. Providence, RI: Brown University; 2008. Full text: exclude #2.

Mitchell JM. Effects of physician-owned limited-service hospitals: evidence from Arizona. Health Aff (Millwood). 2005;Suppl Web Exclusives:W5-481-90. Full text: exclude #2.

Mitchell JM. Utilization changes following market entry by physician-owned specialty hospitals. Medical care research and review : MCRR. 2007;64(4):395-415. Full text: exclude #2.

Popovic JR. 1999 National Hospital Discharge Survey: annual summary with detailed diagnosis and procedure data. Vital Health Stat. 2001;13(151):i-v, 1-206. Full text: exclude #2.

Rivers PA, Fottler MD, Younis MZ. Does certificate of need really contain hospital costs in the United States? Health Education Journal. 2007;66(3):229-44. Full text: exclude #3

Ross JS, Ho V, Wang Y, Cha SS, Epstein AJ, Masoudi FA, et al. Certificate of need regulation and cardiac catheterization appropriateness after acute myocardial infarction. Circulation. 2007;115(8):1012-9. Full text: exclude #3

State of California Office of Statewide Health Planning and Development. California hospital risk-adjusted mortality rates, coronary artery bypass graft (CABG) surgery, 2003-2011. Sacramento, CA: California Health and Human Services; 2014. Full text: exclude
